# Nitrogen acquisition strategy and its effects on invasiveness of a subtropical invasive plant

**DOI:** 10.3389/fpls.2023.1243849

**Published:** 2023-08-21

**Authors:** Ming Guan, Xiao-Cui Pan, Jian-Kun Sun, Ji-Xin Chen, De-Liang Kong, Yu-Long Feng

**Affiliations:** ^1^ Liaoning Key Laboratory for Biological Invasions and Global Changes, College of Bioscience and Biotechnology, Shenyang Agricultural University, Shenyang, Liaoning, China; ^2^ Zhejiang Provincial Key Laboratory of Plant Evolutionary Ecology and Conservation, School of Life Sciences, Taizhou University, Taizhou, Zhejiang, China; ^3^ College of Forestry, Henan Agricultural University, Zhengzhou, Henan, China

**Keywords:** exotic plant invasion, nitrogen form preference, nitrogen levels, ^15^N labeling, plant nitrogen form acquisition strategy, plasticity in nitrogen form uptake

## Abstract

**Introduction:**

Preference and plasticity in nitrogen (N) form uptake are the main strategies with which plants absorb soil N. However, little effort has been made to explore effects of N form acquisition strategies, especially the plasticity, on invasiveness of exotic plants, although many studies have determined the effects of N levels (e.g. N deposition).

**Methods:**

To address this problem, we studied the differences in N form acquisition strategies between the invasive plant *Solidago canadensis* and its co-occurring native plant *Artemisia lavandulaefolia*, effects of soil N environments, and the relationship between N form acquisition strategy of *S. canadensis* and its invasiveness using a ^15^N-labeling technique in three habitats at four field sites.

**Results:**

Total biomass, root biomass, and the uptakes of soil dissolved inorganic N (DIN) per quadrat were higher for the invasive relative to the native species in all three habitats. The invader always preferred dominant soil N forms: NH_4_^+^ in habitats with NH_4_^+^ as the dominant DIN and NO_3_^-^ in habitats with NO_3_^-^ as the dominant DIN, while *A. lavandulaefolia* consistently preferred NO_3_^-^ in all habitats. Plasticity in N form uptake was higher in the invasive relative to the native species, especially in the farmland. Plant N form acquisition strategy was influenced by both DIN levels and the proportions of different N forms (NO_3_^-^/NH_4_^+^) as judged by their negative effects on the proportional contributions of NH_4_^+^ to plant N (*f*_NH4_^+^) and the preference for NH_4_^+^ (*β*_NH4_^+^). In addition, total biomass was positively associated with *f*_NH4_^+^ or *β*_NH4_^+^ for *S. canadensis*, while negatively for *A. lavandulaefolia*. Interestingly, the species may prefer to absorb NH_4_^+^ when soil DIN and/or NO_3_^-^/NH_4_^+^ ratio were low, and root to shoot ratio may be affected by plant nutrient status per se, rather than by soil nutrient availability.

**Discussion:**

Our results indicate that the superior N form acquisition strategy of the invader contributes to its higher N uptake, and therefore to its invasiveness in different habitats, improving our understanding of invasiveness of exotic plants in diverse habitats in terms of utilization of different N forms.

## 1 Introduction

Invasions by exotic plant species can not only severely affect species composition, structure, and function of invaded ecosystems, but also pose a serious threat to the social economy (Chen et al., 2016; Kerr et al., 2016; Iqbal et al., 2020; Kumar Rai and Singh, 2020; Zhao et al., 2020). Many studies have focused on understanding how exotic plants successfully invade new environments, and how to predict and prevent exotic plant invasions (Catford et al., 2009; Lau and Schultheis, 2015; Enders et al., 2020; Huang et al., 2020; Liu et al., 2022). It is generally believed that high competitiveness and adaptability to new environments contribute to successful invasion of exotic plants (Blossey and Notzold, 1995; Feng et al., 2009; Liao et al., 2020; Zheng et al., 2020). The efficient absorption and utilization of soil nitrogen (N) is one of the key functional traits that endow invasive plants with competitive advantages (Castro-Díez et al., 2014; Parepa et al., 2019; Huang et al., 2020; Liu et al., 2022; Luo et al., 2022; Guo et al., 2023). Thus, understanding how invasive plants gain advantages in soil N uptake over natives can provide an important scientific basis for the effective prediction and prevention of exotic plant invasions.

Plants can directly absorb nitrate (NO_3_^-^), ammonium (NH_4_^+^) and N-containing organic micromolecules such as amino acids from soils (McKane et al., 2002; Houle et al., 2014; Sun et al., 2021). However, different plant species have different abilities to absorb these N forms due to many reasons, for example their contents and proportions in soils, differences in their mobility in soils (Brady and Weil, 1999) and energy consumption when assimilated in cells (Salsac et al., 1987), and the interspecific differences in expressions of various N transport genes for absorbing different N forms (Luo et al., 2022; Zhang et al., 2022a), sensitivities to NH_4_^+^ toxicity (Britto and Kronzucker, 2002; Zhang et al., 2022b), and associations with symbiotic microorganisms. Some plants show preferences for a particular form of soil N, regardless of the availability of alternative N forms (Huangfu et al., 2016; Chen and Chen, 2018; Tang et al., 2020; Luo et al., 2022; Zhang et al., 2022a). For example, Rice (*Oryza sativa*), *Xanthium sibiricum* and invasive plant *Flaveria bidentis* prefer to absorb NH_4_^+^, while wheat (*Triticum aestivum*), the invasive plant *X. strumarium* and *Ipomoea cairica* prefer to absorb NO_3_^-^ (Li et al., 2013; Huangfu et al., 2016; Chen and Chen, 2018; Luo et al., 2022). Some plants can adjust their uptake of different N forms according to their proportions in soil, i.e., showing plasticity in N form uptake (Andersen and Turner, 2013; Russo et al., 2013; Sun et al., 2021). It has been found that plants have different absorption capacities and preferences for different soil N forms in different habitats (Averill and Finzi, 2011; Wang and Macko, 2011; Boczulak et al., 2014). Compared with the plants that always prefer a specific N form in different habitats, plants with plasticity in N form uptake may have advantage in N acquisition, contributing to increasing their competitiveness and making them superior competitors. Numerous studies have demonstrated that the main soil N form (Its content is higher than those of others) varies in different habitats (Wilson et al., 2005; Zhang et al., 2013). Ammonium is the main N form in infertile or acidic (especially hypoxic) soils (Wilson et al., 2005; Zhang et al., 2013), while NO_3_^-^ in fertile aerated or alkaline (including neutral) soils (Wilson et al., 2005). However, few studies have investigated the main soil N forms, N form acquisition strategies, and their relationship for a given plant in different habitats.

Like superior competitors in alpine tundra (Ashton et al., 2010), alpine meadow (Song et al., 2015), and subalpine coniferous forest (Zhang et al., 2018), invasive plants may have higher plasticity in N form uptake than co-occurring natives, or preferentially utilize the main soil N form in different habitats. If so, the invaders will be better adapted to the variations in N sources within and across various habitats, and will be able to acquire more quantities of soil N. Such N uptake strategies can give invasive plants a competitive advantage over natives, promoting their successful invasion. However, few studies have focused on the plasticity in N form uptake of invasive plants. The habitats of invasive plants are diverse, and the contents and relative proportions of NH_4_^+^ and NO_3_^-^ in soils exhibit a high degree of spatial and temporal heterogeneity (Andersen and Turner, 2013). The heterogeneity in soil N forms and the differences in plant N form acquisition strategies may inevitably affect the distribution of invasive plants, and the expression of their invasiveness (Yu and He, 2021a; Yu and He, 2021b). However, very few studies have explored the impacts of the contents and proportions of different soil N forms on N form acquisition strategies of invasive plants, and their relationships with their successful invasion.

*Solidago canadensis*, native to North America, is a highly invasive and destructive weed in many countries. It is now widely distributed throughout the eastern and southern provinces of China. *S. canadensis* has caused serious damage to native ecosystems and economic development (Lu et al., 2005; Li et al., 2016b). A previous study has shown that *S. canadensis* grows larger and has greater chlorophyll content, higher root biomass allocation and stronger low-N tolerance than its congeneric native species under different NO_3_^-^/NH_4_^+^ ratios and levels (Yu and He, 2021a). However, it is unclear whether or how the N form acquisition strategy of *S. canadensis* changes with varying soil N levels and the proportions of different N forms, and how these characteristics affect its invasiveness.

In this study, we measured the contents and the proportions of different N forms in rhizosphere soils of *S. canadensis* and its co-occurring native plant *Artemisia lavandulaefolia*, and their N form acquisition strategies using ^15^N-labelling technique. In order to increase the variations in soil N contents and the proportions of different N forms, this study was conducted in three habitats (farmland, wasteland, and roadside) at four sites, where *S. canadensis* invades seriously. The main purposes of this study were to explore: (1) the differences in N form acquisition strategies between *S. canadensis* and *A. lavandulaefolia* in different habitats; (2) the effects of the variations in soil N contents and the proportions of different N forms on N form acquisition strategies of the invasive and native plants; and (3) the effects of N form acquisition strategy of *S. canadensis* on its invasiveness. We hypothesize that compared with the native plant the invader may have higher ability to adjust their absorption of different N forms according to their availability in soils, i.e., showing higher plasticity in N form uptake, and thus absorb more N in each habitat, contributing to its invasiveness. This study is significant for understanding the effects of N acquisition strategies on invasion success of exotic plants, and also provides a theoretical basis for predicting future spread of invasive plants, and making strategies to manage them.

## 2 Materials and methods

### 2.1 Study sites

Our study was conducted in August of 2020 at four sites in Zhejiang Province, east China: Ningbo (29°54′ N, 121°26′ E; 4 m asl), Xiangshan (29°22′ N, 121°45′ E; 135 m asl), Taizhou (28°52′ N, 120°55′ E; 211 m asl), and Wenzhou (27°56′ N, 120°42′ E; 5 m asl). These sites were all heavily invaded by *S. canadensis*. There is a typical subtropical monsoon climate in these sites, with a mean annual temperature (MAT) of 16°C – 19°C, and a mean annual precipitation (MAP) of 1200 – 1900 mm. In each site, farmland, wasteland, and roadside were chosen as study habitats, where soil N contents and the proportions of different N forms may be different (Li et al., 2014; Zhou et al., 2015; Zhao et al., 2017). The farmlands in our study sites were planted with *Ipomoea batatas* or *Brassica napus*, and all were invaded by *S. canadensis*. At the wasteland and roadside habitats in the four sites, we selected herbaceous communities with less human interference, in which the dominant native plants mainly included *A. lavandulaefolia*, *Setaria viridis*, *Paspalum thunbergii*, *Humulus scandens*, *Geranium carolinianum*, and *Ranunculus cantoniensis*. We found numerous patches of coexisting *S. canadensis* and *A. lavandulaefolia* in the three habitats of the four study sites during a field survey. We selected *A. lavandulaefolia* as the native plant to compare with *S. canadensis* for the following reasons: (1) Both belong to the Asteraceae family, sharing similar evolutionary history; (2) more importantly, they commonly co-occur in the wild in southern China (EBFC, 1985). According to the local residents, *S. canadensis* began to invade in the four areas in 2005. The characteristics of rhizosphere soils of *S. canadensis* and *A. lavandulaefolia* in the three habitats of the four sites are summarized in **Table S1**.

At each habitat in each study site, three 1.0 m × 1.0 m quadrats (> 5 m apart from one another) were randomly established, where the coverage of *S. canadensis* was greater than 90%. Nearby each *S. canadensis* quadrat, we established a 1.0 m × 1.0 m quadrat with more than 90% coverage of *A. lavandulaefolia*. The paired quadrats of *S. canadensis* and *A. lavandulaefolia* within each habitat were less than 5 m apart from each other in order to ensure similar soil physico-chemical properties.

### 2.2 ^15^N labeling and sample collection

Three individuals of *S. canadensis* or *A. lavandulaefolia* (> 15 cm apart from one another) with similar size were selected for ^15^N labeling in each quadrat, and one for each of the three N treatments: ^15^NH_4_^+^, ^15^NO_3_^-^, and control. The ^15^N-labeled ammonium chloride (NH_4_Cl, ^15^N 99.12 atom%) and sodium nitrate (NaNO_3_, ^15^N 99.21 atom%) were purchased from Shanghai Engineering Research Center for Stable Isotopes (Shanghai, China). Each plant for the control treatment was treated with 48 mL deionized water with no N addition. A given mass of ^15^NH_4_Cl and ^15^NaNO_3_ (containing 360 μg ^15^N) was weighed, dissolved in 48 mL deionized water (0.5 mmol ^15^N L^-1^), and applied for each individual plant. The nitrification inhibitor dicyandiamide (DCD) was added to each sampled plant (75 mg plant^-1^; corresponding to ≈50 µg g^-1^ soil) in order to prevent potential ammonium oxidation (Zhu et al., 2019). To ensure homogeneous distribution of the labeling solutions in the soil around each labeled plant, we used the Rhizon Cera soil solution sampler (Rhizosphere Research Products, Wageningen, Netherlands) instead of a traditional sterile syringe needle to inject the isotopic solution.

The front of the sampler is a 10-cm long porous polyester tube, with a diameter of 5 mm and many uniform pores of 0.15 μm. This sampler could release the labeling solution or deionized water evenly into different parts of the soil when pressure is carefully applied to the syringe. The effectiveness of the sampler had been confirmed in our preliminary experiments using trypan blue dye. We further determined the minimal number of the samplers needed, the volume of the solution needed to add into each sampler, and its insertion depth into soil in order to achieve a homogeneous distribution of the solution in the soil around each labeled plant. Based on these preliminary experiments, the labeling method was as follows: carefully removing plant litter from soil surface around each target plant, and putting a circular injection template on the ground with the plant as the center (**Figure S1**). The injection template was a hardboard circle (11 cm in diameter), which matches the outside diameter of the Luoyang shovel. On the template, a circle with a radius of 2.5 cm was drawn and six holes (0.5 bore diameter) were made evenly along the circumference. Then we drilled six holes into the soil up to 10 cm depth around the target plant, inserted the sampler with 8 mL labeling solution into each hole to the depth of 10 cm, and finally injected the solution into soil. Using this method, the solution was evenly dispersed in the soil inside a cylinder with a height of 15 cm and a radius of 5 cm centered around the plant.

Forty-eight hours after ^15^N labeling, plant material and rhizosphere soil were collected for each labeled or control plant. We first clipped each plant at 1 cm above ground, then dug out the soil (including roots; not necessary to collect all roots of the plant, just the roots within the range of ^15^N labeling) around the plant with a radius of 5 cm and to a depth of 15 cm using a specialized soil auger (Luoyang shovel, 10 cm in internal diameter). The shoot and soil of each plant were immediately put into plastic self-sealing bags, respectively, which were stored in an ice box. The plant and soil samples collected every day were transported back to our laboratory on the same day. Rhizosphere soil for each soil sample was collected using a hand-shaking method in the laboratory (Zhao et al., 2020), passed through a 2-mm sieve, and separated into two portions. One portion (≈10 g) was air-dried at room temperature for determination of total N and C contents, while the other portion was stored at 4°C for determination of NH_4_^+^ and NO_3_^-^ contents. The roots in each soil sample were collected, rinsed immediately with water, soaked in 0.5 mmol L^-1^ CaCl_2_ solution for 5 min, and then rinsed thoroughly with deionized water to remove the ^15^N adsorbed on the root surface (Cui et al., 2017). The roots and the shoot from each sample plant were oven-dried at 60°C to constant weight, and then ground to a fine powder for determination of total N and δ^15^N contents using a ball mill (GT200, Grinder, China) with 1400 r min^-1^ for 30 s.

### 2.3 Measurements

#### 2.3.1 Plant biomass and root to shoot ratio

In the mono-dominant community of *S. canadensis* or *A. lavandulaefolia* at each habitat in each study site, three quadrats (0.5 m × 0.5 m) were randomly established for biomass measurement. The above-ground plant tissues (stems and leaves) in each quadrat were clipped above ground surface, and put into a kraft paper bag. Roots were carefully dug out with a shovel (to a depth of 15 cm; more than 95% of the total roots), shaken to remove soil, rinsed with water, and then put into a kraft paper bag. Shoots and roots were transported to our laboratory, oven-dried to constant weight at 60°C, and weighed using an analytical balance, respectively for each quadrat. Total above- and belowground biomass (g m^-2^) were calculated per square meter, and root to shoot ratio was calculated for each quadrat.

#### 2.3.2 Total plant N concentration and δ^15^N

Total N concentration and δ^15^N in the whole plant powder were measured using an element analyzer-stable isotopic mass spectrometer (Flash EA 1112 HT-Delta V Advantage, Thermo Fisher Scientific, Waltham, MA, USA). The measurement was conducted by the Third Institute of Oceanography, Ministry of Natural Resources, Xiamen, China. Three compounds were used as references: DL-alanine (δ^15^N = -1.7‰), glycine (δ^15^N = 10‰), and histidine (δ^15^N = -8‰). The analytical precision for δ^15^N was 0.2‰.

#### 2.3.3 Soil dissolved inorganic N content

Ten gram of each rhizosphere soil sample was weighed accurately, extracted in 50 mL 2 mol L^-1^ KCl using a reciprocal shaker (200 r min^-1^ for 1 h), and then filtered through Whatman #1 filter paper. The concentrations of NH_4_^+^ and NO_3_^-^ was determined using an Auto Analyzer III instrument (AA3, SEAL Analytical, Norderstedt, Germany).

### 2.4 Calculations

#### 2.4.1 Plant uptake of different N forms

The ^15^N atom% excess of the labeled plant compared with that of the control plant (APE_labeled_, %) was calculated according to McKane et al. (2002) and Cui et al. (2017) as follows:


(1) $$APE_{labeled}=\;AT\%\;excess_{labeled}-\;AT\%\;excess_{control}=\;AT{\%}_{labeled}-\;AT{\%}_{control}\;$$

where AT% excess_labeled_ or AT% excess_control_ indicates the difference in the ^15^N atom% between the labeled (AT%_labeled_, ^15^N/(^15^N + ^14^N) × 100) or the control plant (AT%_control_) and the atmosphere (^15^N AT%_atm_). Uptake of ^15^N by the labeled plant (^15^N_uptake_, μg) was calculated as follows:


(2) $$15^{\;}N_{uptake}=\;APE_{labeled}\times \;total\;biomass\;\times \;N_{content}\times \;1000\;=\;\left[\left(AT{\%}_{labeled}-\;AT{\%}_{control}\right)/100\right]\;\times \;{\left(total\;biomass\;\times \;N_{content}\right)}_{labeled}\times \;1000\;\;\;\;\;\;\;$$

where, total biomass is the sum of above- and underground biomass of the labeled plant (g), and N_content_ is the N content of the labeled plant (%). The ^15^N uptake rate of the plant (^15^N_uptake_ rate, μg N g^-1^ root h^-1^) was calculated as follows: 


(3) $$15^{\;}N_{uptake}rate\;{=}^{15}N_{uptake}/\left(root\;biomass\;\times \;time\right)\;\;\;$$

where time is the duration of labeling treatment (h), and root biomass was in gram. The uptake for the existing N (either ^14^N or ^15^N) in soil by the labeled plant (Actual N uptake) was calculated according to McKane et al. (2002) and Zhang et al. (2019) as follows: 


(4) $$Actual\;N\;uptake\;{=}^{15}N_{uptake}\times \;C_{available/}C^{15}N_{added}\;\;\;\;$$

where C_available_ is the content of the existing NO_3_^-^ or NH_4_^+^ in the soil (mg N kg^-1^ dw soil), and C^15^N_added_ is the content of the ^15^N-NO_3_^-^ or ^15^N-NH_4_^+^ added into the soil (mg N kg^-1^ dw soil). The uptake rate of the labeled plant for the existing NO_3_^-^ or NH_4_^+^ in the soil (Actual N uptake rate, μg N g^-1^ root h^-1^) was calculated as follows: 


(5) $$Actual\;N\;uptake\;rate\;=\;{[}^{15}{N_{uptake}\times \;C_{available/}C^{15}N_{added}]}_{/}[root\;biomass\;\times \;time]\;{=}^{15}N_{uptake}rate\times \;C_{available/}C^{15}N_{added}\;\;\;\;\;\;\;\;\;\;\;$$

The uptake for the NO_3_^-^ or NH_4_^+^ that already presented in the soil before N labeling treatment by the plants in each quadrat (N Uptake per quadrat, μg m^-2^) was calculated as follows:


(6) $$Uptake\;per\;quadrat\;=\;Actual\;N\;uptake\;rate\;\times \;root\;biomass_{quadrat}\times \;time$$

where root biomass_quadrat_ is the sum of root biomass in each quadrat (g m^-2^).

The proportional contribution of NO_3_^-^ (*f*_NO3_^-^) or NH_4_^+^ (*f*_NH4_^+^) to plant N was calculated as the fraction of the actual uptake rate of NO_3_^-^ or NH_4_^+^ in the total actual uptake rate of NO_3_^-^ and NH_4_^+^ (Guo et al., 2021).

#### 2.4.2 Plant N form preference

Plant preferences (*β*) for different inorganic N forms were calculated according to Liu et al. (2013) and Zhang et al. (2018) as follows:


(7) $$\beta_{NF}=f_{NF}-\;\left[NF\right]/\left[DIN\right].$$

Where *β*_NF_, *f*_NF_, [NF]/[DIN] were the preference for a certain inorganic N form, the proportional contribution of this N form to plant N, and the proportional contribution of this N form to DIN (NH_4_^+^ and NO_3_^-^) of the soil, respectively. *β*_NF_ > 0 indicates a preference for this N form; *β*_NF_< 0 indicates no preference for this inorganic N form, but a preference for the other inorganic N form; and *β*_NF_ = 0 indicates no preference.

#### 2.4.3 Plasticity in plant N form uptake

Based on McKane et al. (2002); Kahmen et al. (2006), and Gao et al. (2020), the percentage similarity between plant uptake of different N forms and the availability of those N forms in rhizosphere soil (PS or percentage similarity) was used to estimate the plasticity of plant N form uptake, which was calculated as follows:


(8) $$PS\;\left(\%\right)\;=\;100\;–\;0.5\;\times \;[(|f_{NH4}{+}^{\;}–\;[NH_{4}{+}^{\;}]\;/\;[DIN]\left|)\;+\;(\right|f_{NO3}{-}^{\;}–\;[NO_{3}{-}^{\;}]\;/\;[DIN]\left|\right)]\;\times \;100]$$

The higher the value of the percentage similarity, the greater the plant plasticity in the inorganic N form uptake. The value of the percentage similarity = 100% indicates that the plant absorbs the two N forms strictly according to their proportions in rhizosphere soil, i.e., that the plant absorbs soil inorganic N absolutely using the plastic strategy. The lower the value of the percentage similarity, the lower the plant plasticity in the inorganic N form uptake, indicating a preference or negative preference for specific N form.

### 2.5 Statistical analysis

Linear mixed-effects model was conducted to test the effects of habitats, species, and their interactions on each variable. Habitats, species, and their interactions were used as fixed factors, and quadrats nested within study sites as random factors. The models were performed in R (version 4.2.2) using the ‘lme’ and ‘anova.lme’ functions of the ‘nlme’ package (Pinheiro et al., 2016). One-way analysis of variance (ANOVA) was conducted to detect the difference in each variable for the same species (*S. canadensis* or *A. lavandulaefolia*) among different habitats. Independent samples *t*-test was used to detect the difference in each variable between *S. canadensis* and *A. lavandulaefolia* in the same habitat, and the difference between *β*_NF_ and 0. These analyses were carried out using SPSS (version 2018; SPSS Inc., Chicago, IL, USA). The relationships between the values of *f*_NH4_^+^ or *β*_NH4_^+^ versus soil DIN contents or the ratios of NO_3_^-^ to NH_4_^+^, and those between total biomass or root to shoot ratios versus the values of *f*_NH4_^+^ or *β*_NH4_^+^ were analyzed for each species with standardized major axis (SMA) regression, using the ‘smatr’ package in R (Warton et al., 2012). We first tested whether the slopes of SMA regressions were significantly different between *S. canadensis* and *A. lavandulaefolia*; if not, we further tested the interspecific differences in intercepts and the shift along the common slope. Before all above-mentioned analyses, the preferences for different soil N forms were quantile-transformed, and the other variables were *log*-transformed in order to meet the assumptions of normality (Shapiro-Wilk tests) and homoscedasticity (Levene’s test). Linear regression analysis was used to examine the significance of the correlations between root to shoot ratios versus total biomass, and those between the contents of total dissolved inorganic nitrogen, NO_3_^-^ and NH_4_^+^ versus root to shoot ratios for each species.

## 3 Results

### 3.1 Total biomass, root biomass, and root to shoot ratio

**Figure 1:**
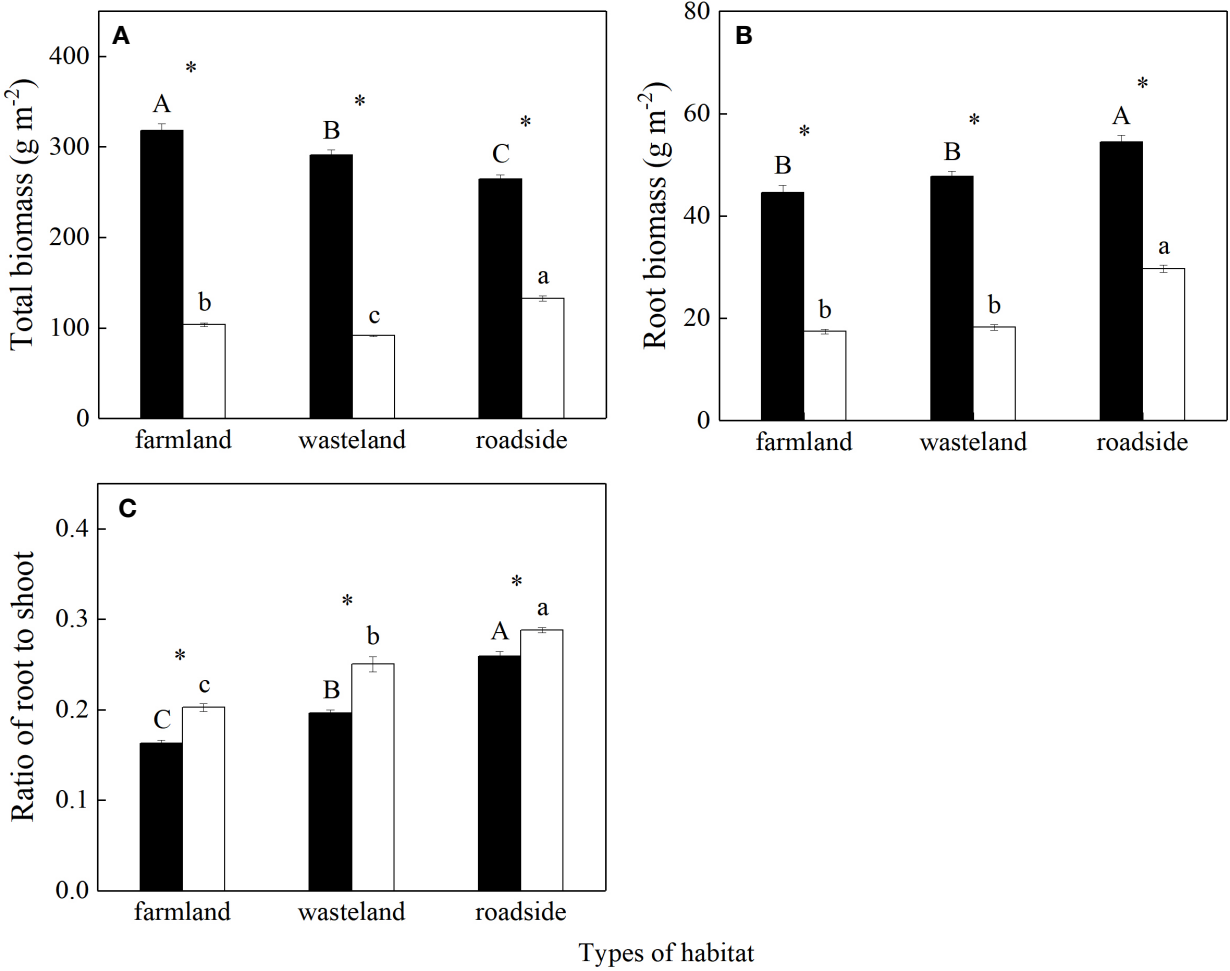
Total biomass **(A)**, root biomass **(B)**, and root to shoot ratio **(C)** of *Solidago canadensis* (closed bars) and *Artemisia lavanduiaefolia* (open bars) in different habitats. Mean ± SE (*n* = 12). Different upper- and lowercase letters indicate significant differences among habitats for *S. canadensis* and *A*. *lavanduiaefolia*, respectively (*P*< 0.05; one-way ANOVA); * indicates significant differences between the two species in the same habitat (*P*< 0.05; independent sample *t*-test).

Total biomass, root biomass, and root to shoot ratio were significantly affected by habitats, species, and their interactions (*P*< 0.05; **Table S2**). Total biomass of *S. canadensis* was the highest in the farmland, and the lowest in the roadside (*P*< 0.05; **Figure 1A**). In contrast, total biomass of *A. lavandulaefolia* was the highest in the roadside, and the lowest in the wasteland (*P*< 0.05). Total biomass were significantly higher for the invasive relative to the native species in all three habitats (*P*< 0.05).

For both species, root biomass was similar in the farmland and wasteland, both significantly lower than that in the roadside (*P*< 0.05; **Figure 1B**). Root biomass was significantly higher for the invasive relative to the native species in all three habitats (*P*< 0.05).

For both species, root to shoot ratios were the highest in the roadside, and the lowest in the farmland (*P*< 0.05; **Figure 1C**). Root to shoot ratios were significantly lower for the invasive relative to the native species in all three habitats (*P*< 0.05).

### 3.2 Contents of different N forms in rhizosphere soils

**Figure 2:**
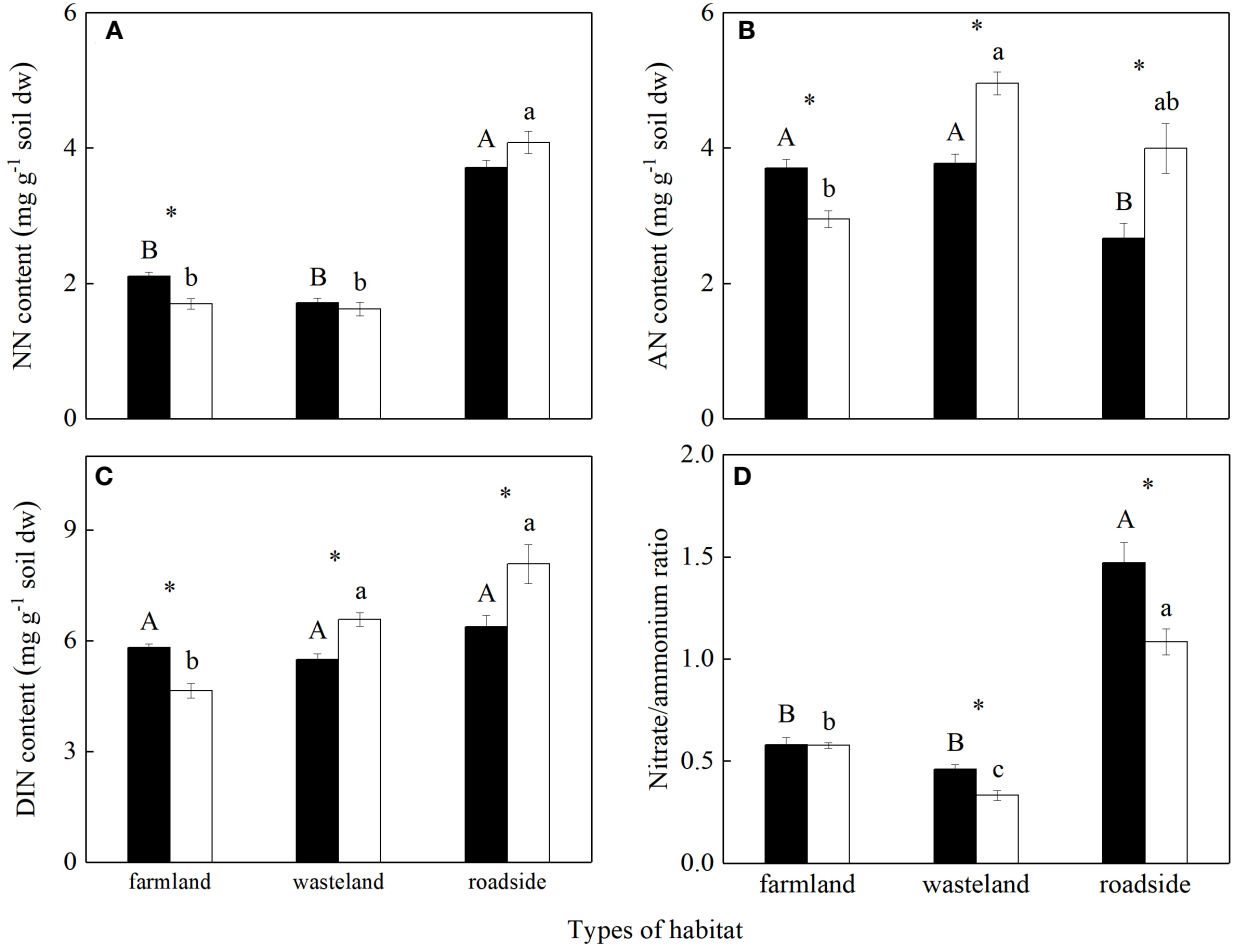
Contents of NO_3_^-^ **(A)**, NH_4_^+^ **(B)** and total dissolved inorganic nitrogen **(C)**, and the ratio of NO_3_^-^ to NH_4_^+^ **(D)** in the rhizosphere soils of *Solidago canadensis* (closed bars) and *Artemisia lavanduiaefolia* (open bars) in different habitats. NN, nitrate nitrogen; AN, ammonium nitrogen; DIN, dissolved inorganic nitrogen. Mean ± SE (*n* = 12). Different upper- and lowercase letters indicate significant differences among habitats for *S. canadensis* and *A*. *lavanduiaefolia*, respectively (*P*< 0.05; one-way ANOVA); * indicates significant differences between the two species in the same habitat (*P*< 0.05; independent sample *t*-test).

The contents of NO_3_^-^, NH_4_^+^ and DIN in rhizosphere soil, and the ratio of NO_3_^-^ to NH_4_^+^ were all significantly influenced by habitats, species, and their interactions (*P*< 0.05; **Table S3**). For both the invasive and native species, soil NO_3_^-^ contents were similar in the farmland and wasteland, both significantly lower than that in the roadside (*P*< 0.05; **Figure 2A**). The invader was significantly higher in soil NO_3_^-^ content than *A. lavandulaefolia* in the farmland (*P*< 0.05), but similar in the wasteland and roadside.

For *S. canadensis*, NH_4_^+^ contents in rhizosphere soils were similar in the farmland and wasteland, both significantly higher than that in the roadside (*P*< 0.05; **Figure 2B**). For *A. lavandulaefolia*, soil NH_4_^+^ content was significantly higher in the wasteland than in the farmland (*P*< 0.05), which were not significantly different with that in the roadside (*P* > 0.05). The invader was significantly higher in soil NH_4_^+^ content than *A. lavandulaefolia* in the farmland, while lower in the wasteland and roadside (*P*< 0.05).

For *S. canadensis*, DIN contents in rhizosphere soils were similar in all three habitats (*P* > 0.05; **Figure 2C**). For *A. lavandulaefolia*, soil DIN contents were similar in the roadside and wasteland, both significantly higher than that in the farmland (*P*< 0.05). Similarly as in soil NH_4_^+^ content, the invader was significantly higher in soil DIN content than *A. lavandulaefolia* in the farmland, while lower in the wasteland and roadside (*P*< 0.05).

For *S. canadensis*, the ratios of NO_3_^-^ to NH_4_^+^ in rhizosphere soils were similar in the farmland and wasteland, both significantly lower than that in the roadside (*P*< 0.05; **Figure 2D**). For *A. lavandulaefolia*, the ratio of NO_3_^-^ to NH_4_^+^ was the highest in the roadside, followed by the farmland and wasteland, respectively (*P*< 0.05). Compared with *A. lavandulaefolia*, *S. canadensis* showed a significantly higher ratio of NO_3_^-^ to NH_4_^+^ in the wasteland and roadside (*P*< 0.05), but not in the farmland (*P* > 0.05).

### 3.3 Uptakes for different N forms in rhizosphere soils

**Figure 3:**
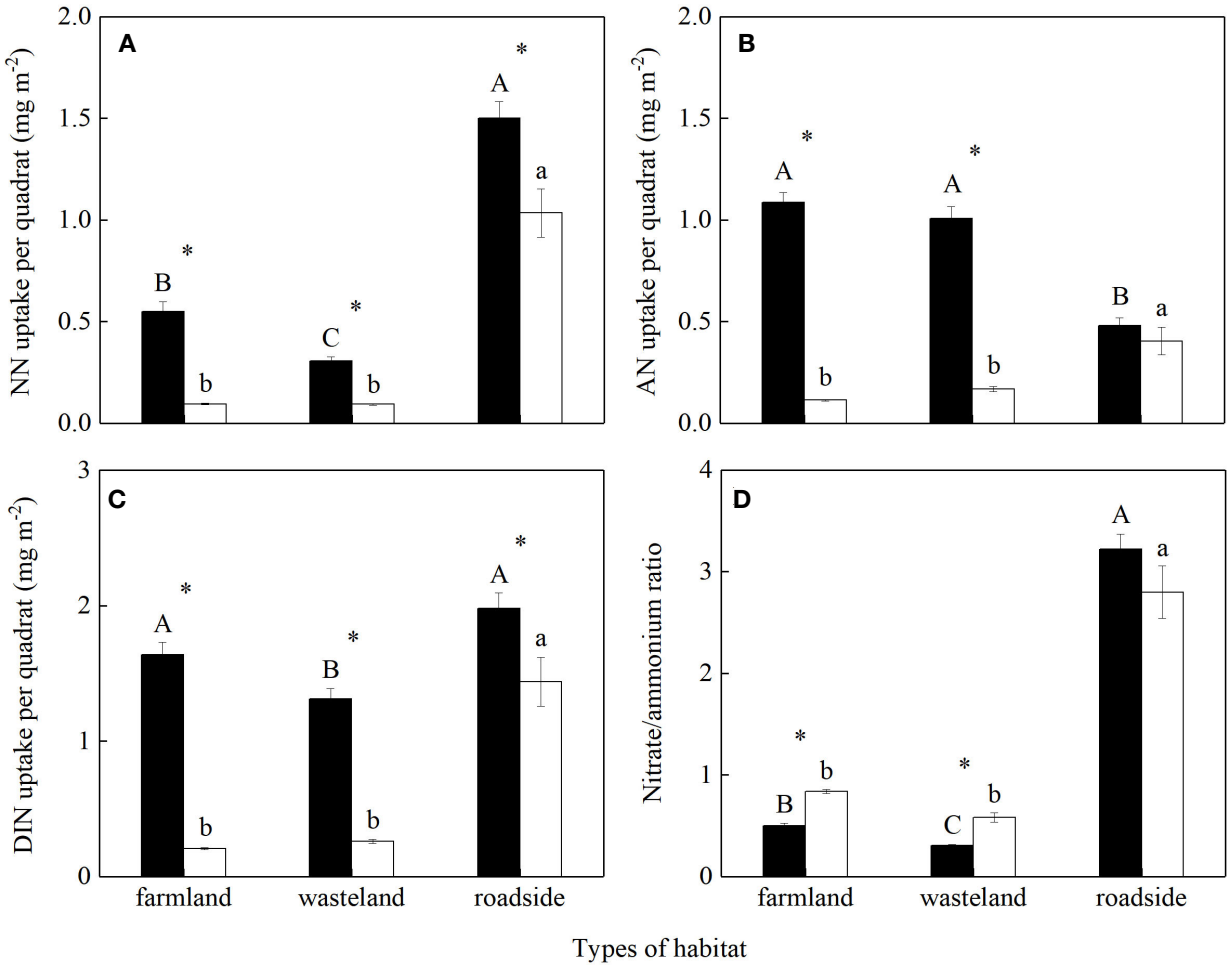
Uptakes of NO_3_^-^ **(A)**, NH_4_^+^ **(B)** and total dissolved inorganic nitrogen **(C)** existing in soil, and the ratio of NO_3_^-^ to NH_4_^+^ **(D)** absorbed by *Solidago canadensis* (closed bars) and *Artemisia lavanduiaefolia* (open bars) in different habitats. NN, nitrate nitrogen; AN, ammonium nitrogen; DIN, dissolved inorganic nitrogen. Mean ± SE (*n* = 12). Different upper- and lowercase letters indicate significant differences among habitats for *S. canadensis* and *A. lavanduiaefolia*, respectively (*P*< 0.05; one-way ANOVA); * indicates significant differences between the two species in the same habitat (*P*< 0.05; independent sample *t*-test).

The uptakes of soil NO_3_^-^, NH_4_^+^ and DIN per quadrat, and the uptake ratio of NO_3_^-^ to NH_4_^+^ were all significantly influenced by habitats, species, and their interactions (*P*< 0.05; **Table S4**). For *S. canadensis*, the uptake of soil NO_3_^-^ per quadrat was the highest in the roadside, followed by the farmland and wasteland, respectively (*P*< 0.05; **Figure 3A**). For *A. lavandulaefolia*, the uptakes of soil NO_3_^-^ per quadrat were similar in the farmland and wasteland, both significantly lower than that in the roadside (*P*< 0.05). The uptakes of soil NO_3_^-^ per quadrat were significantly higher for the invasive relative to the native species in all three habitats (*P*< 0.05).

In the farmland and wasteland compared with the roadside, the uptake of soil NH_4_^+^ per quadrat were significantly higher for *S. canadensis*, while significantly lower for *A. lavandulaefolia* (*P*< 0.05; **Figure 3B**). Compared with *A. lavandulaefolia*, *S. canadensis* showed significantly higher NH_4_^+^ uptake per quadrat in the farmland and wasteland (*P*< 0.05), but not in the roadside (*P* > 0.05).

For *S. canadensis*, the uptakes of soil DIN per quadrat were similar in the farmland and roadside, both significantly higher than that in the wasteland (*P*< 0.05; **Figure 3C**). For *A. lavandulaefolia*, the uptakes of soil DIN per quadrat were similar in the farmland and wasteland, both significantly lower than that in the roadside (*P*< 0.05). The uptakes of soil DIN per quadrat were significantly higher for the invasive relative to the native species in all three habitats (*P*< 0.05).

For *S. canadensis*, the uptake ratios of soil NO_3_^-^ to NH_4_^+^ was highest in the roadside, followed by the farmland and wasteland, respectively (*P*< 0.05; **Figure 3D**). For *A. lavandulaefolia*, the uptake ratios of NO_3_^-^ to NH_4_^+^ were similar in the farmland and wasteland, both significantly lower than that in the roadside (*P*< 0.05). Compared with *A. lavandulaefolia*, *S. canadensis* showed significantly lower uptake ratios of NO_3_^-^ to NH_4_^+^ in the farmland and wasteland (*P*< 0.05), but not in the roadside (*P* > 0.05).

### 3.4 Proportional contribution of different N forms to plant N

**Figure 4:**
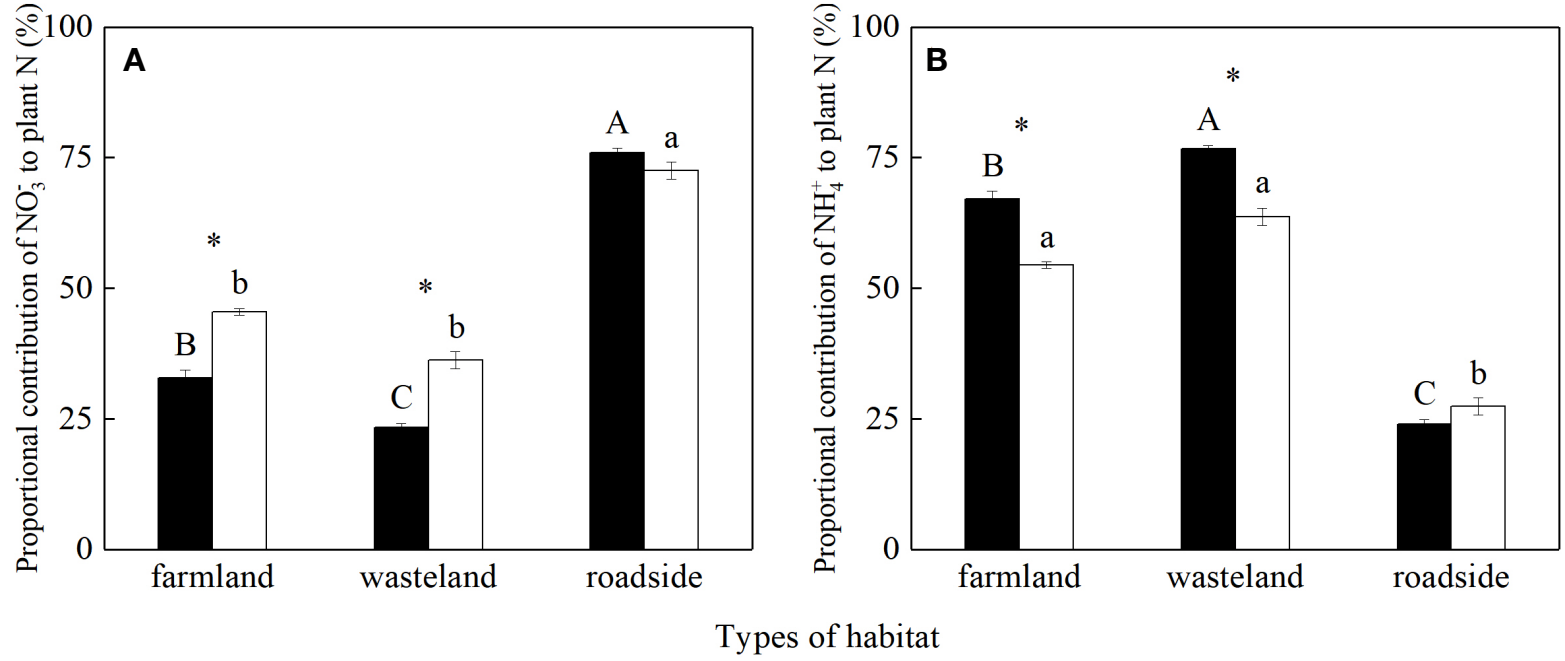
Proportional contributions (%) of soil NO_3_^-^ **(A)** and NH_4_^+^ **(B)** to plant N of *Solidago canadensis* (closed bars) and *Artemisia lavanduiaefolia* (open bars) in different habitats. Mean ± SE (*n* = 12). Different upper- and lowercase letters indicate significant differences among habitats for *S. canadensis* and *A. lavanduiaefolia*, respectively (*P*< 0.05; one-way ANOVA); * indicates significant differences between the two species in the same habitat (*P*< 0.05; independent sample *t*-test).

The values of *f*_NO3_^-^ and *f*_NH4_^+^ were significantly influenced by habitats, species, and their interactions (*P*< 0.05; **Table S6**). For *S. canadensis*, the value of *f*_NO3_^-^ was the highest in the roadside, followed by the farmland and wasteland, respectively (*P*< 0.05; **Figure 4A**). For *A. lavandulaefolia*, the values of *f*_NO3_^-^ were similar in the farmland and wasteland, both significantly lower than that in the roadside (*P*< 0.05). Compared with *A. lavandulaefolia*, *S. canadensis* showed significantly lower *f*_NO3_^-^ values in the farmland and wasteland (*P*< 0.05), but not in the roadside (*P* > 0.05).

For *S. canadensis*, the value of *f*_NH4_^+^ was the highest in the wasteland, followed by the farmland and roadside, respectively *(P*< 0.05; **Figure 4B**). For *A. lavandulaefolia*, the values of *f*_NH4_^+^ were similar in the farmland and wasteland, both significantly higher than that in the roadside (*P*< 0.05). Compared with *A. lavandulaefolia*, *S. canadensis* showed significantly higher *f*_NH4_^+^ values in the farmland and wasteland (*P*< 0.05), but not in the roadside (*P*< 0.05).

### 3.5 Preference for different N forms

For *S. canadensis*, there was no significant difference between *β*_NO3_^-^ or *β*_NH4_^+^ versus zero in the farmland (*P* > 0.05), indicating no significant preference for N forms; in the wasteland the value of *β*_NO3_^-^ was significantly lower than zero and the value of *β*_NH4_^+^ was significantly higher than zero, indicating a preference for NH_4_^+^; and in the roadside the value of *β*_NO3_^-^ was significantly higher than zero and the value of *β*_NH4_^+^ was significantly lower than zero, showing a preference for NO_3_^-^. For *A. lavandulaefolia*, the values of *β*_NO3_^-^ were significantly higher than zero in all three habitats, while the values of *β*_NH4_^+^ were significantly lower than zero, indicating a consistent preference for NO_3_^-^.

**Figure 5:**
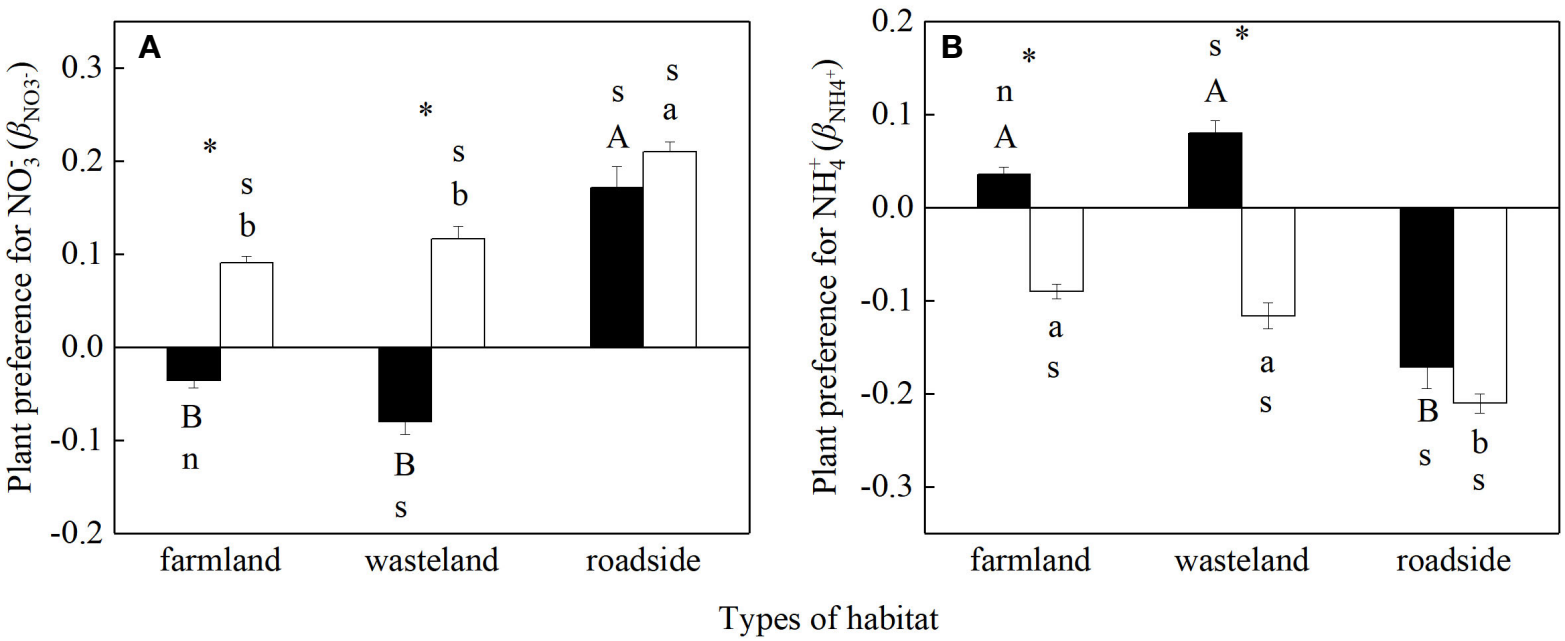
Preference for NO_3_^-^ **(A)** and NH_4_^+^ **(B)** for *Solidago canadensis* (closed bars) and *Artemisia lavanduiaefolia* (open bars) in different habitats. Mean ± SE (*n* = 12). s and n indicate significant and non-significant differences with 0, respectively (*P*< 0.05; independent sample *t*-test). Different upper- and lowercase letters indicate significant differences among habitats for *S. canadensis* and *A. lavanduiaefolia*, respectively (*P*< 0.05; one-way ANOVA); * indicates significant differences between the two species in the same habitat (*P*< 0.05; independent sample *t*-test).

The values of *β*_NO3_^-^ and *β*_NH4_^+^ were significantly influenced by habitats, species, and their interactions (*P*< 0.05; **Table S7**). For both species, the values of *β*_NO3_^-^ were similar in the farmland and wasteland, both significantly lower than that in the roadside (*P*< 0.05; **Figure 5A**). For the values of *β*_NH4_^+^, however, both species were significantly lower in the roadside than in the farmland and wasteland (*P*< 0.05; **Figure 5B**).

Compared with *A. lavandulaefolia*, *S. canadensis* showed significantly lower values of *β*_NO3_^-^ and significantly higher values of *β*_NH4_^+^ in the farmland and wasteland (*P*< 0.05). There was no significant difference in the values of *β*_NO3_^-^ and *β*_NH4_^+^ between the two species in the roadside (*P* > 0.05).

### 3.6 Plasticity in plant uptake for different N form

The percentage similarity between plant uptake patterns of different N forms and their pattern of availability in rhizosphere soil was significantly influenced by habitats and species (*P*< 0.05; **Table S8**), while the effect of the interaction of these factors was not significant (*P* = 0.798).

**Figure 6:**
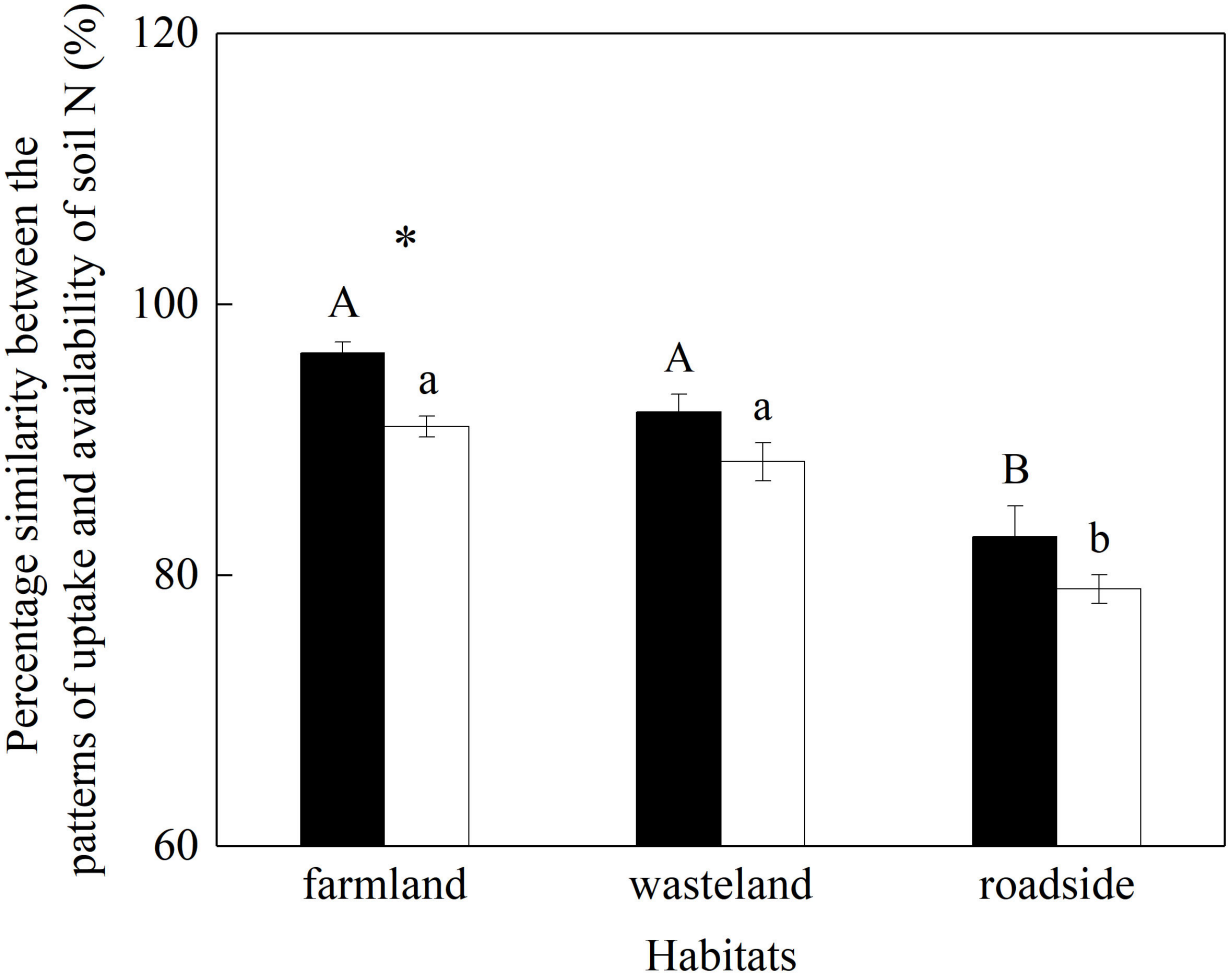
Percentage similarity between plant uptake pattern of different N forms and their pattern of availability in rhizosphere soil of *Solidago canadensis* (closed bars) and *Artemisia lavanduiaefolia* (open bars) in different habitats. Mean ± SE (*n* = 12). Different upper- and lowercase letters indicate significant differences among habitats for *S. canadensis* and *A. lavanduiaefolia*, respectively (*P*< 0.05; one-way ANOVA); * indicates significant differences between the two species in the same habitat (*P*< 0.05; independent sample *t*-test).

For both species, the vales of percentage similarity were similar in the farmland and wasteland, both significantly higher than that in the roadside (*P*< 0.05; **Figure 6**). Compared with *A. lavandulaefolia*, *S. canadensis* showed significantly higher value of percentage similarity in the farmland (*P*< 0.05), but not in the wasteland and roadside(*P* > 0.05).

### 3.7 Effects of soil DIN contents and NO_3_^-^/NH_4_^+^ ratios on *f*_NH4_^+^ and *β*_NH4_^+^

**Figure 7:**
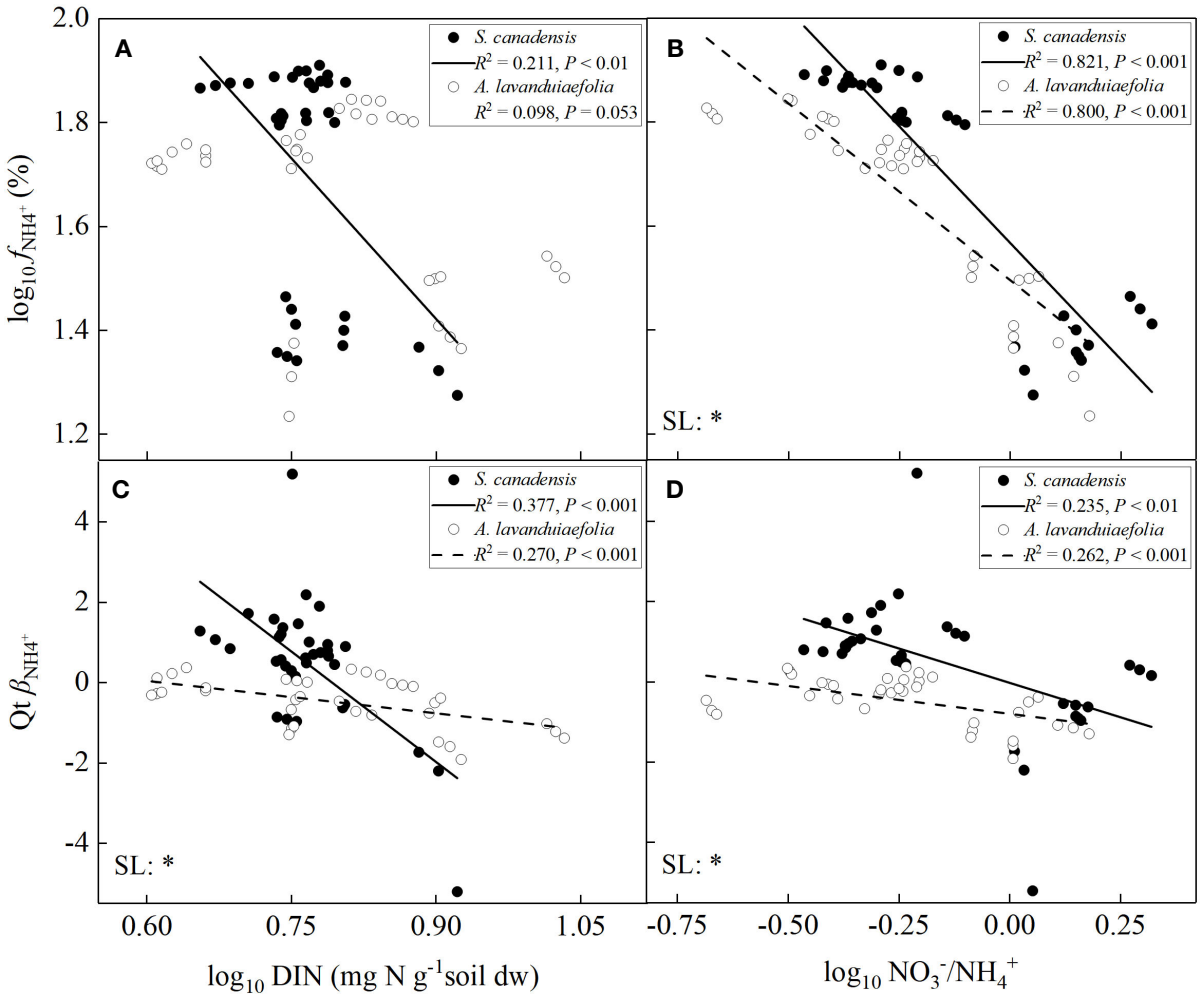
Relationships between *f*_NH4_^+^ **(A, B)** and *β*_NH4_^+^ **(C, D)** versus total soil dissolved inorganic nitrogen contents and the ratios of NO_3_^-^ to NH_4_^+^ for *Solidago canadensis* and *Artemisia lavanduiaefolia*, respectively. Only significant SMA lines are shown (*R* ^2^ > 0.1, *P*< 0.05). DIN, dissolved inorganic nitrogen. SL, slope; *, significant differences.

For both the invasive and native species, the values of *f*_NH4_^+^ or *β*_NH4_^+^ decreased significantly with increasing soil DIN contents or the ratios of NO_3_^-^ to NH_4_^+^ except the values of *f*_NH4_^+^ with soil DIN contents for *A. lavandulaefolia* (marginally significant) (*P*< 0.05; **Figure 7**). The SMA slope of the relationship between the values of *β*_NH4_^+^ and soil DIN contents was significantly lower for *S. canadensis* than for *A. lavandulaefolia* (*P*< 0.05), indicating that the values of *β*_NH4_^+^ was more strongly influenced by change in soil DIN contents for the invasive relative to the native species. The SMA slopes of the relationship between the values of *f*_NH4_^+^ or *β*_NH4_^+^ and the ratios of NO_3_^-^ to NH_4_^+^ were also significantly lower for *S. canadensis* than for *A. lavandulaefolia* (*P*< 0.05), indicating that the values of *f*_NH4_^+^ and *β*_NH4_^+^ were more strongly influenced by change in the ratios of NO_3_^-^ to NH_4_^+^ for the invasive relative to the native species.

### 3.8 Effects of *f*_NH4_^+^ and *β*_NH4_^+^ on total biomass and root/shoot ratios

**Figure 8:**
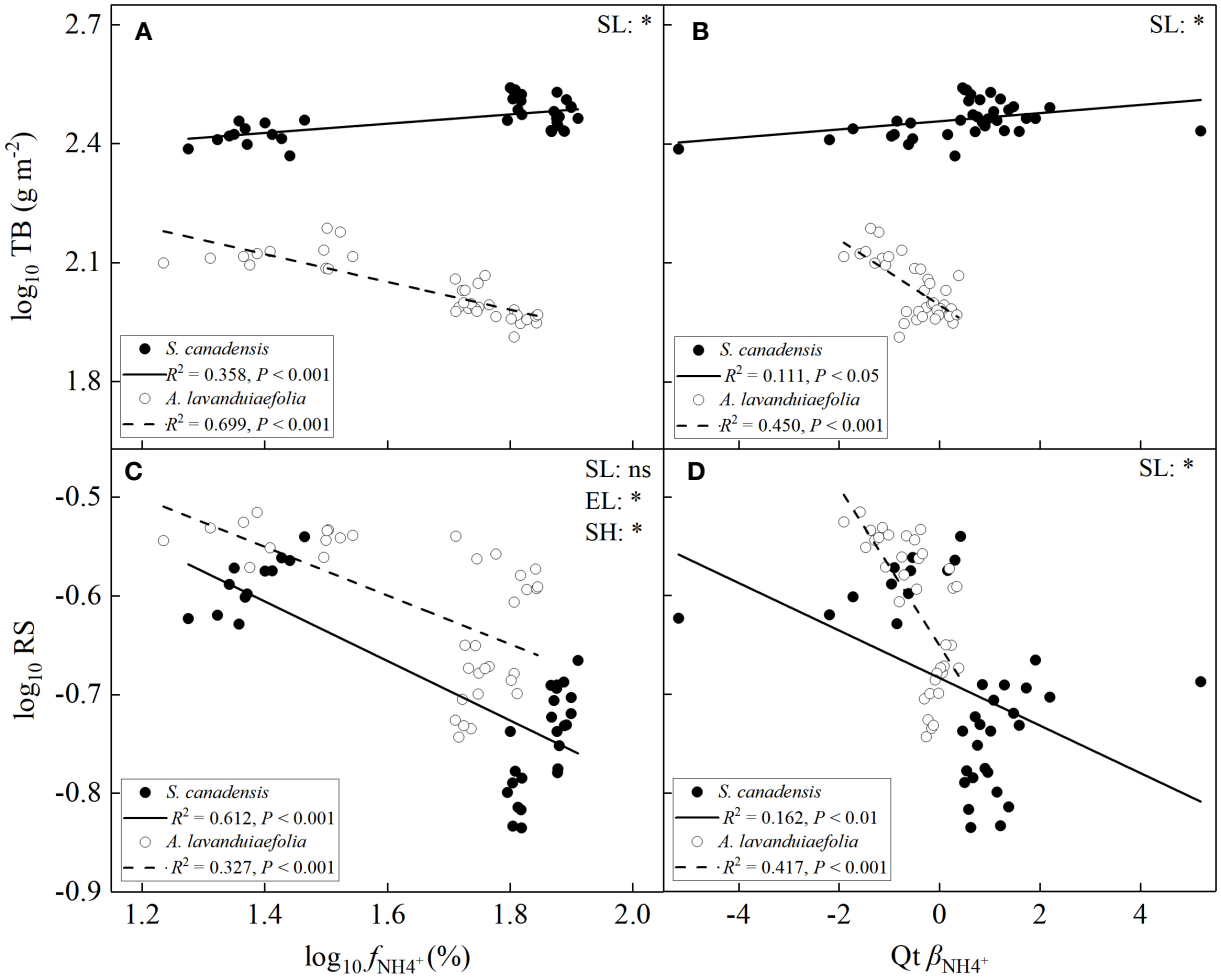
Relationships between total biomass **(A, B)** and root to shoot ratios **(C, D)** versus *f*_NH4_^+^ and *β*_NH4_^+^ for *Solidago canadensis* and *Artemisia lavanduiaefolia*, respectively. Only significant SMA lines are shown (*R* ^2^ > 0.1, *P*< 0.05). TB, total biomass; RS, root to shoot ratio; SL, slope; EL, elevation or intercept; SH, shift along common slope. *, significant differences; ns, not significant differences.

Total biomass increased significantly with the increase of the values of *f*_NH4_^+^ or *β*_NH4_^+^ for *S. canadensis* (*P*< 0.001; **Figure 8A, B**), while decreased significantly for *A. lavandulaefolia*. The absolute values of the SMA slope of the relationship were significantly lower for *S. canadensis* than for *A. lavandulaefolia*.

For both species, the root to shoot ratios significantly decreased with the increase of the values of *f*_NH4_^+^ or *β*_NH4_^+^ (*P*< 0.01; **Figure 8C, D**). The SMA slopes of the relationships between root to shoot ratios and the values of *f*_NH4_^+^ were not significantly different between the two species (*P* > 0.05). The value of the *y*-intercept of the relationship was significantly higher for *A. lavandulaefolia* than for *S. canadensis* (*P*< 0.05), indicating that root to shoot ratio was significantly lower in *S. canadensis* than in *A. lavandulaefolia* under the same value of *f*_NH4_^+^. The shift along the common slope of the relationship was also significantly different between the two plants (*P*< 0.05), with *S. canadensis* showing higher values of *f*_NH4_^+^ but lower root to shoot ratios, and *A. lavandulaefolia* showing lower values of *f*_NH4_^+^ but higher root to shoot ratios. The SMA slope of the relationship between root to shoot ratios and the values of *β*_NH4_^+^ was significantly higher for *S. canadensis* than for *A. lavandulaefolia* (*P*< 0.05), indicating that root to shoot ratios were less influenced by the change in the values of *β*_NH4_^+^ for the invasive relative to the native species.

## 4 Discussion

Consistent with our hypothesis, the invasive plant *S. canadensis* absorbed more N than the native plant *A. lavandulaefolia* in all three habitats, contributing to its invasion success as judged by its significantly higher total biomass. Numerous studies have demonstrated that N is one of the vital factors that influences invasion success of exotic plants (Lee et al., 2012; Castro-Díez et al., 2014; Sun et al., 2021). Compared with native plants, invasive plants not only have stronger abilities to absorb soil N, and higher leaf N contents (Huang et al., 2020; Liu et al., 2022), but also utilize leaf N more efficiently (Feng et al, 2009; Feng et al, 2011). Feng et al. (2009) found that the invasive relative to native populations of *Ageratina adenophora* allocate lower leaf N to cell walls but higher to photosynthetic organs, resulting in higher photosynthetic rates and N-use efficiencies. In addition, we further found that N form acquisition strategies of the invasive and native species were influenced by both soil N levels and the proportions of different N forms (**Figure 7**). More importantly, our results provided robust evidence for the association between N form acquisition strategy of *S. canadensis* and its invasiveness.

### 4.1 N form acquisition strategy and exotic plant invasiveness

In the farmland and wasteland, the invader had both higher DIN uptake rates (**Figure S2C**) and total root biomass per quadrat (**Figure 1B**), both contributing to its higher N uptake. James et al. (2009) also found that N uptake rates were significantly higher in three invasive perennial forbs than in six native perennial grasses and forbs in both heterogeneous and homogeneous nutrient soils. In the roadside, however, the higher total root biomass per quadrat was the main reason for the higher N uptake of the invader, where its DIN uptake rate was lower than that of *A. lavandulaefolia* (**Figure S2C**). Of course, we do not know whether the invader could absorb more organic N than *A. lavandulaefolia* in the roadside, which warrants further study. A recent study showed that *S. canadensis* could absorb organic N, particularly in habitats rich in free amino acids (Yu et al., 2016). Similarly, for *S. canadensis*, total biomass was the lowest in the roadside among the habitats (**Figure 1A**), while the uptake of soil DIN in the roadside was similar with that in the farmland, and higher than that in the wasteland. These results indicate that the invader may also absorb organic N in the wasteland and farmland. In general, the organic N content in the farmland is higher than that in the wasteland and roadside (Lv et al., 2011; Quan et al., 2022). Specific root morphology, high carbon allocation to roots, and a flexible N uptake strategy may all contribute to the high N uptake rates of invasive plants (James et al., 2009; Hewins and Hyatt, 2010; Mozdzer et al., 2010; Hu et al., 2019). In our study, the higher plasticity in soil N form uptake and the preference for the dominant soil N form contributed to the higher N uptake rate of *S. canadensis* (see below). In addition, the invasive relative to the native species also showed more stable N uptake rates across all three habitats (**Figure S2C**). The stability of DIN uptake may contribute to adaptation of the invader to temporal and spatial fluctuations in soil N availability, and thus to invasion success of the invader in the different habitats.

The higher total root biomass of the invasive relative to the native species was due to its faster growth (i.e., higher total biomass), rather than to the interspecific difference in root to shoot ratio. The invader had significantly lower root to shoot ratios in all three habitats. Lower root to shoot ratios were also found in other invasive species relative to their co-occurring natives (Zou et al., 2007; te Beest et al., 2009; Liao et al., 2013; Liao et al., 2019). The low root to shoot ratios may contribute to invasiveness of exotic species in fertile habitats, by leaving more biomass for allocation to shoot and thus increasing utilization of aboveground resources. Negative relationship between total biomass and root to shoot ratios was indeed found for the invader (**Figure S3A**). This result also indicates that the invader had allometric growth relationship between root and shoot.

Root to shoot ratio was not influenced by soil nutrient levels (DIN contents) for *S. canadensis* (**Figure S4**), which is different with the results of many other studies (Liao et al., 2013; Guo et al., 2019; Yan et al., 2019; Li et al., 2020). Liao et al. (2013) found that root to shoot ratio of the invasive plant *Chromolaena odarata* decreased significantly with increasing soil nutrient in both mono- and mixed cultures. Addition of N also decreases root to shoot ratio of *Arabidopsis thaliana* (Yan et al., 2019). However, root to shoot ratio of the invader decreased significantly with increasing soil NH_4_^+^ content, while increased with increasing soil NO_3_^-^ content (**Figure S4**). The invader can better absorb NH_4_^+^ compared with NO_3_^-^ (see below), and thus increasing soil NH_4_^+^ can better improve its N status. These results indicate that root to shoot ratio of the invader may be influenced by its nutrient status, rather than by soil nutrient levels per se. Root to shoot ratios were negatively correlated with *f*_NH4_^+^ or *β*_NH4_^+^ for both the invasive and native species (**Figure 8C, D**). Along the common SMA slope of the two species, *S. canadensis* was located at the end with low root to shoot ratios and high *f*_NH4_^+^ values. This result indicates that the higher *f*_NH4_^+^ was at least one of the reasons for the lower root to shoot ratio for the invasive species. Consistently, the values of *f*_NH4_^+^ were significantly higher (**Figure 4**), while root to shoot ratios lower for the invader in the farmland and wasteland than in the roadside (**Figure 1**).

Consistent with our hypothesis, the invasive relative to the native species had higher plasticity in uptake of different soil N forms, contributing to its more DIN uptake. In the farmland and wasteland, where NH_4_^+^ was the dominant DIN in rhizosphere soils for both species, the invasive and native species absorbed NH_4_^+^ relative to NO_3_^-^ more quickly, and thus NH_4_^+^ contributed more greatly to plant N. In the roadside, where NO_3_^-^ was the dominant DIN in rhizosphere soils for the two species, both species absorbed NO_3_^-^ relative to NH_4_^+^ more quickly, and thus NO_3_^-^ contributed more greatly to plant N. These results indicate that the invasive and native species had plasticity in N form uptake. This plasticity could ensure that the two species always utilized the dominant soil N form, and thus contributed to their adaptation to the changes in soil N forms. The higher plasticity in N form uptake for the invasive relative to the native species (especially in the farmland) could help the invader to adapt to the changes in soil N forms. Plasticity in N form uptake have also been found in other plants (Andersen and Turner, 2013; Russo et al., 2013). For example, some plants switch their N source from NO_3_^-^ to NH_4_^+^ when their habitats change from dry to wet (Houlton et al., 2007; Wang and Macko, 2011). Plasticity in N form uptake may be a basic strategy for plants to adapt to the changes in soil N forms (Ashton et al., 2010), and an important factor determining plant dominances and diversity patterns (Craine and Dybzinski, 2013). Until now, however, very few references have studied the roles of plasticity in N form uptake in exotic plant invasions, especially using a quantitative estimator.

Preferential uptake of N forms also contributed to the more N uptake of the invasive relative to the native species. In the farmland and wasteland, the invader preferred NH_4_^+^, especially in the wasteland, and the N (DIN) uptake rates of the invader were significantly higher than those of the native species (**Figure S2C**). The higher DIN uptake rates were mainly associated with its higher NH_4_^+^ uptake rates, while its NO_3_^-^ uptake rate was not significantly higher than that of *A. lavandulaefolia* in the wasteland. In the roadside, where NO_3_^-^ was the dominant soil N, the invader preferred NO_3_^-^. however, *A. lavandulaefolia* always preferred NO_3_^-^ in three habitats. These results indicate that the invader could adjust its preference for N form according to the dominant soil N form, while *A. lavandulaefolia* could not. The invader always preferred to absorb the dominant soil N form, contributing to its higher N uptake, and therefore to its invasiveness.

We indeed found that total biomass was positively associated with *f*_NH4_^+^ or *β*_NH4_^+^ for the invader, while the relationships were negative for *A. lavandulaefolia* (**Figure 8**). This result indicates that increasing preference for NH_4_^+^ and its proportional contribution to plant N increased invasiveness of the invader. A previous study also found that *S. canadensis* grows better in soils with a higher ratio of NH_4_^+^ to NO_3_^-^ soils, indicating its preference for NH_4_^+^ (Lu et al., 2005). Preferential uptake of N forms was also found in other plants (Huangfu et al., 2016; Chen and Chen, 2018; Tang et al., 2020; Luo et al., 2022; Zhang et al., 2022a). Luo et al. (2022) found that preference for NO_3_^-^ relative to NH_4_^+^ may help the invasive plant *X. strumarium* to invade NO_3_^–^enriched disturbed habitats. However, the reasons for the difference in the preference for soil N forms between invasive and native species are still poorly understood.

### 4.2 Factors affecting plant N form acquisition strategy

Our results showed that plant N form acquisition strategy was influenced by both soil N levels and the proportions of different N forms (**Figure 7**). Numerous studies have shown that the habitats invaded by exotic plants are diverse, and the levels and the proportions of NO_3_^-^ and NH_4_^+^ in these habitats are different greatly (Peng et al., 2011; Andersen and Turner, 2013; Li et al., 2014; Li et al., 2016a; Wang et al., 2020). However, few studies have investigated the effects of these factors on plant N form uptake strategy for invasive plants. We found that *S. canadensis* and *A. lavandulaefolia* increased their preferences for NH_4_^+^ and the proportional contributions of NH_4_^+^ to plant N with decreasing soil DIN contents and the ratios of NO_3_^-^ to NH_4_^+^. These results indicate that plants are more likely to prefer NH_4_^+^ and NH_4_^+^ is the main N source for plants in barren relative to fertile habitats or in habitats with low relative to high ratios of NO_3_^-^ to NH_4_^+^. However, the values of *f*_NH4_^+^ and *β*_NH4_^+^ were more susceptible to the changes in soil DIN contents and the ratios of NO_3_^-^ to NH_4_^+^ for the invasive relative to the native species, indicating that the invader responded more sensitively to the changes in soil contents of NO_3_^-^ to NH_4_^+^ and their ratios (**Figure 7**). In addition, the values of *f*_NH4_^+^ and *β*_NH4_^+^ were significantly higher for the invasive relative to the native species in habitats with low DIN contents or low ratios of NO_3_^-^ to NH_4_^+^.

Plants absorb NH_4_^+^ and NO_3_^-^ using different N transporters, and the differences in the expressions of the genes of these transporters may explain interspecific difference in N form preference (genetic basis). For example, many NO_3_^-^ and NH_4_^+^ transporter genes are significantly different in sequences, or differentially expressed between the invasive plant *X. strumarium* (preference for NO_3_^-^) and its native congener *X. sibiricum* (preference for NH_4_^+^) (Luo et al., 2022; Zhang et al., 2022a).

The differences in sensitivities to NH_4_^+^ toxicity may also contribute to the interspecific differences in N form preference (Britto and Kronzucker, 2002; Niinemets, 2010). Zhang et al. (2022b) found that *X. strumarium* is more sensitive to NH_4_^+^, and always preferred NO_3_^-^, contributing to alleviating NH_4_^+^ toxicity at high levels (Lambers et al., 1998). We do not know whether *A. lavandulaefolia* is more sensitive to NH_4_^+^ than the invader, and whether this is the reason for that pattern *A. lavandulaefolia* preferred NO_3_^-^ in the farmland and wasteland, where NH_4_^+^ was the dominant soil N form. Further studies are needed.

Other factors such as mycorrhizal type, mycorrhizal taxa and the extent of their infection may also affect interspecific differences in N form preference between invasive and native plants. A better understanding of the degree to which mycorrhizal fungi affect plant N form preferences could significantly improve our understanding of how invasive plant N acquisition strategies will respond to environmental changes.

## 5 Conclusions

The invasive plant *S. canadensis* could adjust preference for N forms according to the variations in the dominant soil N forms, always preferring the dominant soil N form, while the native plant *A. lavandulaefolia* consistently preferred NO_3_^-^ in all habitats. The higher plasticity in N form uptake and the preference for the dominant soil N form make the invader to better absorb the dominant soil N forms, contributing to its more stable and more N uptake, and thus to its invasiveness in the different habitats. With increasing the uptake and preference for soil NH_4_^+^, total biomass increased and root to shoot ratio decreased for the invader. Our study provides robust evidence that invasiveness of exotic plants is associated with their N form acquisition strategy, which is influenced by soil N conditions. These results improve our understanding of invasion success of exotic plants in diverse habitats in terms of utilization of different N forms, especially the role of plasticity in N form uptake.

## Data availability statement

The original contributions presented in the study are included in the article/**Supplementary Material**. Further inquiries can be directed to the corresponding author.

## Author contributions

Y-LF, MG and D-LK conceived the ideal and designed methodology. MG and X-CP conducted the experiments, analyzed the data and drafted the manuscript. J-KS and J-XC assisted with soil dissolved inorganic nitrogen analysis. Y-LF and MG critically reviewed and edited the manuscript. All authors contributed to the article and approved the submitted version.

## References

[B1] Andersen K. M. Turner B. L. (2013). Preferences or plasticity in nitrogen acquisition by understorey palms in a tropical montane forest. J. Ecol. 101 (3), 819–825. doi: 10.1111/1365-2745.12070

[B2] Ashton I. W. Miller A. E. Bowman W. D. Suding K. N. (2010). Niche complementarity due to plasticity in resource use: plant partitioning of chemical N forms. Ecology 91 (11), 3252–3260. doi: 10.1890/09-1849.1 21141186

[B3] Averill C. Finzi A. (2011). Increasing plant use of organic nitrogen with elevation is reflected in nitrogen uptake rates and ecosystem δ^15^N. Ecology 92 (4), 883–891. doi: 10.1890/10-0746.1 21661551

[B4] Blossey B. Notzold R. (1995). Evolution of increased competitive ability in invasive nonindigenous plants: a hypothesis. J. Ecol. 83 (5), 887–889. doi: 10.2307/2261425

[B5] Boczulak S. A. Hawkins B. J. Roy R. (2014). Temperature effects on nitrogen form uptake by seedling roots of three contrasting conifers. Tree Physiol. 34 (5), 513–523. doi: 10.1093/treephys/tpu028 24831958

[B6] Brady N. C. Weil R. R. (1999). The nature and properties of soil. 12th Edn (Toronto, ON: Macmillan).

[B7] Britto D. T. Kronzucker H. J. (2002). NH_4_ ^+^ toxicity in higher plants: a critical review. J. Plant Physiol. 159, 567–584. doi: 10.1078/0176-1617-0774

[B8] Castro-Díez P. Godoy O. Alonso A. Gallardo A. Saldaña A. (2014). What explains variation in the impacts of exotic plant invasions on the nitrogen cycle? A meta-analysis. Ecol. Lett. 17 (1), 1–12. doi: 10.1111/ele.12197 24134461

[B9] Catford J. A. Jansson R. Nilsson C. (2009). Reducing redundancy in invasion ecology by integrating hypotheses into a single theoretical framework. Divers. Distrib. 15 (1), 22–40. doi: 10.1111/j.1472-4642.2008.00521.x

[B10] Chen W.-B. Chen B.-M. (2018). Considering the preferences for nitrogen forms by invasive plants: a case study from a hydroponic culture experiment. Weed Res. 59 (1), 49–57. doi: 10.1111/wre.12344

[B11] Chen B.-M. Peng S.-L. Wu X.-P. Wang P.-L. Ma J.-X. (2016). A bibliometric analysis of researches on topics related to the ecological damage caused by and risk assessments of exotic invasive species from 1995 to 2014. Acta Ecol. Sin. 36 (20), 6677–6685. doi: 10.5846/stxb201504060690

[B12] Craine J. M. Dybzinski R. (2013). Mechanisms of plant competition for nutrients, water and light. Funct. Ecol. 27 (4), 833–840. doi: 10.1111/1365-2435.12081

[B13] Cui J.-H. Yu C.-Q. Qiao N. Xu X.-L. Tian Y.-Q. Ouyang H. (2017). Plant preference for NH_4_ ^+^ versus NO_3_ ^–^ at different growth stages in an alpine agroecosystem. Field Crop Res. 201, 192–199. doi: 10.1016/j.fcr.2016.11.009

[B14] EBFC. (1985). Flora of China (Editorial Board of FLORA OF CHINA) (Beijing: Science Press).

[B15] Enders M. Havemann F. Ruland F. Bernard-Verdier M. Catford J. A. Gómez-Aparicio L.. (2020). A conceptual map of invasion biology: Integrating hypotheses into a consensus network. Glob. Ecol. Biogeogr. 29, 978–991. doi: 10.1111/geb.13082 34938151 PMC8647925

[B16] Feng Y.-L. Lei Y.-B. Wang R.-F. Callaway R. M. Valiente-Banuet A. Inderjit. (2009). Evolutionary tradeoffs for nitrogen allocation to photosynthesis versus cell walls in an invasive plant. Proc. Natl. Acad. Sci. U.S.A. 106, 1853–1856. doi: 10.1073/pnas.0808434106 19171910 PMC2644127

[B17] Feng Y.-L. Li Y.-P. Wang R.-F. Callaway R. M. Valiente-Banuet A. Inderjit (2011). A quicker return energy-use strategy by populations of a subtropical invader in the non-native range: a potential mechanism for the evolution of increased competitive ability. J. Ecol. 99, 1116–1123. doi: 10.1111/j.1365-2745.2011.01843.x

[B18] Gao L. Cui X.-Y. Hill P. W. Guo Y.-F. (2020). Uptake of various nitrogen forms by co-existing plant species in temperate and cold-temperate forests in northeast China. Appl. Soil Ecol. 147, 103398. doi: 10.1016/j.apsoil.2019.103398

[B19] Guo X. Hu Y. Ma J.-Y. Wang H. Wang K.-L. Wang T.. (2023). Nitrogen deposition effects on invasive and native plant competition: implications for future invasions. Ecotox. Environ. Safe. 259, 115029. doi: 10.1016/j.ecoenv.2023.115029 37216867

[B20] Guo J.-X. Jia Y.-M. Chen H.-H. Zhang L.-J. Yang J.-C. Zhang J.. (2019). Growth, photosynthesis, and nutrient uptake in wheat are affected by differences in nitrogen levels and forms and potassium supply. Sci. Rep. 9 (1), 1–12. doi: 10.1038/s41598-018-37838-3 30718692 PMC6362105

[B21] Guo W.-J. Zhang Z.-L. Liu Q. Xiao J. Yin H.-J. (2021). Seasonal variations in plant nitrogen acquisition in an ectomycorrhizal alpine forest on the eastern Tibetan Plateau, China. Plant Soil 459 (1-2), 79–91. doi: 10.1007/s11104-020-04644-8

[B22] Hewins D. B. Hyatt L. A. (2010). Flexible n uptake and assimilation mechanisms may assist biological invasion by *Alliaria petiolata* . Biol. Invasions 12, 2639–2647. doi: 10.1007/s10530-009-9671-5

[B23] Houle D. Moore J. D. Ouimet R. Marty C. (2014). Tree species partition N uptake by soil depth in boreal forests. Ecology 95 (5), 1127–1133. doi: 10.1890/14-0191.1 25000744

[B24] Houlton B. Z. Sigman D. M. Schuur E. A. G. Hedin L. O. (2007). A climate-driven switch in plant nitrogen acquisition within tropical forest communities. Proc. Natl. Acad. Sci. U.S.A. 104 (21), 8902–8906. doi: 10.1073/pnas.0609935104 17502607 PMC1885600

[B25] Hu Y.-H. Zhou Y.-L. Gao J.-Q. Zhang X.-Y. Song M.-H. Xu X.-L. (2019). Plasticity of plant N uptake in two native species in response to invasive species. Forests 10. doi: 10.3390/f10121075

[B26] Huang K. Kong D.-L. Lu X.-R. Feng W.-W. Liu M.-C. Feng Y.-L. (2020). Lesser leaf herbivore damage and structural defense and greater nutrient concentrations for invasive alien plants: evidence from 47 pairs of invasive and non-invasive plants. Sci. Total Environ. 723, 137829. doi: 10.1016/j.scitotenv.2020.137829 32203801

[B27] Huangfu C.-H. Li H.-Y. Chen X.-W. Liu H.-M. Wang H. Yang D.-L. (2016). Response of an invasive plant, *Flaveria bidentis*, to nitrogen addition: a test of form-preference uptake. Biol. Invasions 18 (11), 3365–3380. doi: 10.1007/s10530-016-1231-1

[B28] Iqbal M. F. Liu M.-C. Iram A. Feng Y.-L. (2020). ). Effects of the invasive plant *Xanthium strumarium* on diversity of native plant species: A competitive analysis approach in North and Northeast China. PloS One 15, e228476. doi: 10.1371/journal.pone.0228476 PMC7676722 33211690

[B29] James J. Mangold J. Sheley R. Svejcar T. (2009). Root plasticity of native and invasive Great Basin species in response to soil nitrogen heterogeneity. Plant Ecol. 202 (2), 211–220. doi: 10.1007/sl1258-008-9457-3

[B30] Kahmen A. Renker C. Unsicker S. B. Buchmann N. (2006). Niche complementarity for nitrogen: an explanation for the biodiversity and ecosystem functioning relationship? Ecology 87 (5), 1244–1255. doi: 10.1890/0012-9658(2006)87[1244:NCFNAE]2.0.CO;2 16761603

[B31] Kerr N. Z. Baxter P. W. J. Salguero Gómez R. Wardle G. M. Buckley Y. M. (2016). Prioritizing management actions for invasive populations using cost, efficacy, demography and expert opinion for 14 plant species world-wide. J. Appl. Ecol. 53 (2), 305–316. doi: 10.1111/1365-2664.12592 27478205 PMC4949517

[B32] Kumar Rai P. Singh J. S. (2020). Invasive alien plant species: Their impact on environment, ecosystem services and human health. Ecol. Indic. 111, 106020. doi: 10.1016/j.ecolind.2019.106020 32372880 PMC7194640

[B33] Lambers H. Chapin F. S. Pons T. L. (1998). Plant physiological ecology. (New York, NY: Springer).

[B34] Lau J. A. Schultheis E. H. (2015). When two invasion hypotheses are better than one. New Phytol. 205 (3), 958–960. doi: 10.1111/nph.13260 25580651

[B35] Lee M. R. Flory S. L. Phillips R. P. (2012). Positive feedbacks to growth of an invasive grass through alteration of nitrogen cycling. Oecologia 170, 457–465. doi: 10.1007/s00442-012-2309-9 22526935

[B36] Li J.-M. Du L.-S. Guan W.-B. Yu F.-H. van Kleunen M. (2016b). Latitudinal and longitudinal clines of phenotypic plasticity in the invasive herb *Solidago canadensis* in China. Oecologia 182 (3), 755–764. doi: 10.1007/s00442-016-3699-x 27522606

[B37] Li W.-H. Feng L. Zhou X.-Y. Yue M.-F. Tian X.-S. (2014). Study on distribution of alien invasive weeds and their characteristics of soil NH_4_ ^+^/NO_3_ ^-^ Nitrogen. J. South. Chin. Norm. Univ: Nat. Sci. Ed. 46 (5), 105–111. doi: 10.6054/j.scnun.2014.06.036

[B38] Li S.-X. Wang Z.-H. Stewart B. A. (2013). Responses of crop plants to ammonium and nitrate N. Adv. Agron. 118, 205–397. doi: 10.1016/B978-0-12-405942-9.00005-0

[B39] Li P. Yin R.-B. Shang B. Agathokleous E. Zhou H.-M. Feng Z.-Z. (2020). Interactive effects of ozone exposure and nitrogen addition on tree root traits and biomass allocation pattern: An experimental case study and a literature meta-analysis. Sci. Total Environ. 710, 136379. doi: 10.1016/j.scitotenv.2019.136379 31926420

[B40] Li J.-B. Zhang Q.-T. Zhang L.-Y. Tong C. (2016a). Effect of *Spartina alterniflora* invasion sequence on soil carbon and nitrogen distribution in a *CyPerus malaccensis* marsh of the Min River estuary in spring. Acta Ecol. Sin. 36 (12), 3628–3638. doi: 10.5846/stxb201510092040

[B41] Liao Z.-Y. Scheepens J. F. Li Q.-M. Wang W.-B. Feng Y.-L. Zheng Y.-L. (2020). Founder effects, post-introduction evolution and phenotypic plasticity contribute to invasion success of a genetically impoverished invader. Oecologia 192, 105–118. doi: 10.1007/s00442-019-04566-y 31792607

[B42] Liao Z.-Y. Scheepens J. F. Li W.-T. Wang R.-F. Zheng Y.-L. Feng Y.-L. (2019). Biomass reallocation and increased plasticity might contribute to successful invasion of *Chromolaena odorata* . Flora 256, 79–84. doi: 10.1016/j.flora.2019.05.004

[B43] Liao Z.-Y. Zhang R. Barclay G. F. Feng Y.-L. (2013). Differences in competitive ability between plants from nonnative and native populations of a tropical invader relates to adaptive responses in abiotic and biotic environments. PloS One 8, e71767. doi: 10.1371/journal.pone.0071767 23977140 PMC3745391

[B44] Liu M.-C. Dong T.-F. Feng W.-W. Qu B. Kong D.-L. van Kleunen M.. (2022). leaf trait differences between 97 pairs of invasive and native plants across China: effects of identities of both the invasive and native species. Neobiota 71, 1–22. doi: 10.3897/neobiota.71.71385

[B45] Liu X.-Y. Koba K. Makabe A. Li X.-D. Yoh M. Liu C.-Q. (2013). Ammonium first: natural mosses prefer atmospheric ammonium but vary utilization of dissolved organic nitrogen depending on habitat and nitrogen deposition. New Phytol. 199 (2), 407–419. doi: 10.1111/nph.12284 23692546

[B46] Lu J.-Z. Qiu W. Chen J.-K. Li B. (2005). Impact of invasive species on soil properties: Canadian goldenrod (*Solidago canadensis*) as a case study. Biodiversity Sci. 13 (4), 347–356. doi: 10.1360/biodiv.050071

[B47] Luo J.-J. Gao Y.-M. Feng W.-W. Liu M.-C. Qu B. Zhang C.. (2022). Stronger ability to absorb nitrate and associated transporters in the invasive plant *Xanthium strumarium* compared with its native congener. Environ. Exp. Bot. 198, 104851. doi: 10.1016/j.envexpbot.2022.104851

[B48] Lv X.-J. Liu Q. Chen Y.-P. Li L. (2011). Effect of different land use types on soil dissolved organic carbon and nitrogen in Yellow River Delta. Res. Agric. Modernization 32 (4), 505–508. doi: 10.3969/j.issn.1000-0275.2011.04.028

[B49] McKane R. B. Johnson L. C. Shaver G. R. Nadelhoffer K. J. Rastetter E. B. Fry B.. (2002). Resource-based niches provide a basis for plant species diversity and dominance in arctic tundra. Nature 415 (6867), 68–71. doi: 10.1038/415068a 11780117

[B50] Mozdzer T. J. Zieman J. C. McGlathery K. J. (2010). Nitrogen uptake by native and invasive temperate coastal macrophytes: importance of dissolved organic nitrogen. Estuaries Coasts 33, 784–797. doi: 10.1007/s12237-009-9254-9

[B51] Niinemets Ü. (2010). Responses of forest trees to single and multiple environmental stresses from seedlings to mature plants: past stress history, stress interactions, tolerance and acclimation. For. Ecol. Manage. 260 (10), 1623–1639. doi: 10.1016/j.foreco.2010.07.054

[B52] Parepa M. Kahmen A. Werner R. A. Fischer M. Bossdorf O. (2019). Invasive knotweed has greater nitrogen-use efficiency than native plants: evidence from a ^15^N pulse-chasing experiment. Oecologia 191 (2), 389–396. doi: 10.1007/s00442-019-04490-1 31435756

[B53] Peng R.-H. Fang C.-M. Li B. Chen J.-K. (2011). *Spartina alterniflora* invasion increases soil inorganic nitrogen pools through interactions with tidal subsidies in the Yangtze Estuary, China. Oecologia 165 (3), 797–807. doi: 10.1007/s00442-010-1887-7 21203776

[B54] Pinheiro J. Bates D. DebRoy S. Sarkar D. Team, R. C (2016) nlme: linear and nonlinear mixed effects models. R package version 3.1-128. Available at: http://CRAN.R-project.org/package=nlme (Accessed 1 September 2017).

[B55] Quan Z. Liu X.-A. Liu D. (2022). Research progress on soil soluble organic nitrogen. Chin. J. Appl. Ecol. 33 (1), 277–288. doi: 10.13287/j.1001-9332.202201.010 35224951

[B56] Russo S. E. Kochsiek A. Olney J. Thompson L. Miller A. E. Tan S. (2013). Nitrogen uptake strategies of edaphically specialized Bornean tree species. Plant Ecol. 214 (11), 1405–1416. doi: 10.1007/s11258-013-0260-4

[B57] Salsac L. Chaillou S. Morot-Gaudry J. F. Lesaint C. Jolivoe E. (1987). Nitrate and ammonium nutrition in plants. Plant Physiol. Biochem. 25, 805–812.

[B58] Song M.-H. Zheng L.-L. Suding K. N. Yin T.-F. Yu F.-H. (2015). Plasticity in nitrogen form uptake and preference in response to long-term nitrogen fertilization. Plant Soil 394, 215–224. doi: 10.1007/s11104-015-2532-3

[B59] Sun S.-M. Chen J.-X. Feng W.-W. Zhang C. Huang K. Guan M.. (2021). Plant strategies for nitrogen acquisition and their effects on exotic plant invasions. Biodiversity Sci. 29 (1), 72–80. doi: 10.17520/biods.2020072

[B60] Tang D. Liu M.-Y. Zhang Q. Ma L. Shi Y. Ruan J. (2020). Preferential assimilation of NH_4_ ^+^ over NO_3_ ^–^ in tea plant associated with genes involved in nitrogen transportation, utilization and catechins biosynthesis. Plant Sci. 291, 110369. doi: 10.1016/j.plantsci.2019.110369 31928660

[B61] te Beest M. Stevens N. Olff H. van der Putten W. H. (2009). Plant-soil feedback induces shifts in biomass allocation in the invasive plant *Chromolaena odorata* . J. Ecol. 97 (6), 1281–1290. doi: 10.1111/j.l365-2745.2009.01574.x

[B62] Wang L. Macko S. A. (2011). Constrained preferences in nitrogen uptake across plant species and environments. Plant Cell Environ. 34, 525–534. doi: 10.1111/j.1365-3040.2010.02260.x 21118424

[B63] Wang D. Zhang Y.-R. Feng Y.-L. Liu Z. Qu B. (2020). Changes in vegetation and soil properties following 6 years of enclosure in riparian corridors. J. Plant Ecol. 13 (2), 131–138. doi: 10.1093/jpe/rtaa002

[B64] Warton D. I. Duursma R. A. Falster D. S. Taskinen S. (2012). Smatr 3- an R package for estimation and inference about allometric lines. Methods Ecol. Evol. 3, 257–259. doi: 10.1111/j.2041-210X.2011.00153.x

[B65] Wilson S. M. Pyatt D. G. Ray D. Malcolm D. C. Connolly T. (2005). Indices of soil nitrogen availability for an ecological site classification of british forests. For. Ecol. Manage. 220 (1), 51–65. doi: 10.1016/j.foreco.2005.08.004

[B66] Yan Z.-B. Eziz A. Tian D. Li X.-P. Hou X.-H. Peng H.-Y.. (2019). Biomass allocation in response to nitrogen and phosphorus availability: insight from experimental manipulations of *Arabidopsis thaliana* . Front. Plant Sci. 10. doi: 10.3389/fpls.2019.00598 PMC6528069 31156669

[B67] Yu H.-W. He W.-M. (2021a). Congeneric invasive versus native plants utilize similar inorganic nitrogen forms but have disparate use efficiencies. J. Plant Ecol. 14, 180–190. doi: 10.1093/jpe/rtaa085

[B68] Yu H.-W. He W.-M. (2021b). Plant invaders outperform congeneric natives on amino acids. Basic Appl. Ecol. 54, 75–84. doi: 10.1016/j.baae.2021.04.009

[B69] Yu H.-W. Yang J.-X. Gao Y. He W.-M. (2016). Soil organic nitrogen endows invasive solidago canadensis with greater advantages in low-phosphorus conditions. Ecosphere 7 (3), e01254. doi: 10.1002/ecs2.1254

[B70] Zhang J.-B. Cai Z.-C. Zhu T.-B. Yang W.-Y. Müller C. (2013). Mechanisms for the retention of inorganic N in acidic forest soils of southern China. Sci. Rep. 3. doi: 10.1038/srep02342 PMC3731645 23907561

[B71] Zhang Z.-L. Li N. Xiao J. Zhao C.-Z. Zou T.-T. Li D.-D.. (2018). Changes in plant nitrogen acquisition strategies during the restoration of spruce plantations on the eastern Tibetan Plateau, China. Soil Biol. Biochem. 119, 50–58. doi: 10.1016/j.soilbio.2018.01.002

[B72] Zhang C. Luo J.-J. Zuo J.-B. Zhang Z. Wang S.-T. Zhang X.-J.. (2022a). Transcripts related with ammonium use and effects of gibberellin on expressions of the transcripts responding to ammonium in two invasive and native *Xanthium* species. Front. Plant Sci. 13. doi: 10.3389/fpls.2022.1035137 PMC9644049 36388472

[B73] Zhang Z.-L. Yuan Y.-S. Liu Q. Yin H.-J. (2019). Plant nitrogen acquisition from inorganic and organic sources via root and mycelia pathways in ectomycorrhizal alpine forests. Soil Biol. Biochem. 136, 107517. doi: 10.1016/j.soilbio.2019.06.013

[B74] Zhang Z. Zhang C. Zhang C.-S. Wang W.-B. Feng Y.-L. (2022b). Differences and related physiological mechanisms in effects of ammonium on the invasive plant *Xanthium strumarium* and its native congener *X. sibiricum* . Front. Plant Sci. 13. doi: 10.3389/fpls.2022.999748 PMC9581188 36275581

[B75] Zhao Y.-Z. Liu M.-C. Feng Y.-L. Wang D. Feng W.-W. Clay K.. (2020). Release from below- and aboveground natural enemies contributes to invasion success of a temperate invader. Plant Soil 452, 19–28. doi: 10.1007/s11104-020-04520-5

[B76] Zhao X.-H. Zhang G.-L. Song Z. Zhang R.-H. Yan J. Zhang T.. (2017). Effects of *Solanum rostratum* invasion on soil properties in different soil types. Chin. J. Agrometeorol. 38 (2), 76–87. doi: 10.3969/j.issn.1000-6362.2017.02.002

[B77] Zheng Y.-L. Liao Z.-Y. Li W.-T. Wang R.-F. Li L. Yang A. D.. (2020). The effect of resource pulses on the competitiveness of a tropical invader depends on identity of resident species and resource type. Acta Oecologica 102, 103507. doi: 10.1016/j.actao.2019.103507

[B78] Zhou B. Yan X.-H. Xiao Y.-A. Wang N. Kuang Z.-Q. (2015). Module biomass of *Ageratum conyzoides* populations in different habitats. Acta Ecol. Sin. 35 (8), 2602–2608. doi: 10.5846/stxb201306091493

[B79] Zhu F.-F. Dai L.-M. Hobbie E. A. Koba K. Liu X.-Y. Gurmesa G. A.. (2019). Uptake patterns of glycine, ammonium, and nitrate differ among four common tree species of northeast China. Front. Plant Sci. 10. doi: 10.3389/fpls.2019.00799 PMC6614667 31333684

[B80] Zou J.-W. Roger W. E. Siemann E. (2007). Differences in morphological and physiological traits between native and invasive populations of *Sapium sebiferum* . Funct. Ecol. 21 (4), 721–730. doi: 10.1111/j.1365-2435.2007.01298.x

