# Supplementary material for: Nitrogen acquisition strategy and its effects on invasiveness of a subtropical invasive plant
Source: Front Plant Sci. 2023 Aug 21;14:1243849. doi: 10.3389/fpls.2023.1243849 (PMC10475947; doi:10.3389/fpls.2023.1243849)
Supplement: Supplementary file 1 [file DataSheet_1.docx]

***Supplementary Materials***

**1 Supplementary Figures and Tables**

**1.1 Supplementary Tables**

**Table S1** **Characteristics of rhizosphere soils of *Solidago canadensis* and *Artemisia lavanduiaefolia* in the three habitats of the four study sites**

|  |  | Total N  (mg N g^-1^ dw soil) | Total C  (mg N g^-1^ dw soil) | C: N ratio | Soil pH | LOI (%) |
| --- | --- | --- | --- | --- | --- | --- |
| *S. canadensis* | Farmland | 1.08±0.14A | 18.92±4.31A | 20.42±7.66A | 6.44±0.10C* | 5.36±0.60A* |
|  | Wasteland | 1.37±0.11A | 16.38±1.25A | 12.01±0.34B | 6.88±0.10B* | 5.78±0.39A |
|  | Roadside | 1.23±0.18A | 13.91±1.71A | 11.37±0.79B* | 7.46±0.17A* | 6.08±0.48A |
| *A. lavandulaefolia* | Farmland | 1.2±0.15a | 14.3±1.11a | 12.09±0.52a | 7.41±0.14b | 6.28±0.27a |
|  | Wasteland | 1.47±0.35a | 18.18±2.89a | 12.94±1.95a | 8.05±0.03a | 5.76±0.39a |
|  | Roadside | 2.35±1.03a | 21.93±7.96a | 9.91±0.69a | 8.22±0.06a | 5.55±0.68a |

Note: Mean ± SE (*n* = 12). Different upper-, and lowercase letters indicate significant differences among different habitats for *S. canadensis* and *A. lavanduiaefolia*, respectively (*P* < 0.05; one-way ANOVA); * indicates significant differences between the two species in the same habitat (*P* < 0.05; independent sample *t*-test).

**Table S2 Results of linear mixed-effects model showing the effects of habitats (*n* = 3), species (*n* = 2), and their interactions on total biomass, root biomass, and the ratio of root to shoot.**

| Effect | DF | Total biomass (g m^-2^) | | Root biomass (g m^-2^) | | Ratio of root to shoot | |
| --- | --- | --- | --- | --- | --- | --- | --- |
|  |  | *F* | *P* | *F* | *P* | *F* | *P* |
| Habitats (H) | 2 | 62.17 | **<0.001** | 3.91 | **<0.05** | 40.52 | **<0.001** |
| Species (S) | 1 | 3914.24 | **<0.001** | 1606.37 | **<0.001** | 167.49 | **<0.001** |
| H × S | 2 | 89.98 | **<0.001** | 30.37 | **<0.001** | 7.91 | **<0.001** |

Significant effects (*P* < 0.05) are shown in bold.

**Table S3 Results of linear mixed-effects model showing the effects of habitats (*n* = 3), species (*n* = 2), and their interactions on soil contents of NO_3_^-^, NH_4_^+^ and total dissolved inorganic nitrogen (DIN), and the ratio of NO_3_^-^ to NH_4_^+^.**

| Effect | DF | Soil nitrogen content (mg N kg^-1^ soil dw) | | | | | | Ratio of NO_3_^-^ to NH_4_^+^ | |
| --- | --- | --- | --- | --- | --- | --- | --- | --- | --- |
|  |  | NO_3_^-^ | | NH_4_^+^ | | DIN | |  |  |
|  |  | *F* | *P* | *F* | *P* | *F* | *P* | *F* | *P* |
| Habitats (H) | 2 | 12.38 | **<0.001** | 17.41 | **<0.001** | 9.22 | **<0.01** | 39.60 | **<0.001** |
| Species (S) | 1 | 7.84 | **<0.01** | 60.38 | **<0.001** | 12.12 | **<0.001** | 61.16 | **<0.001** |
| H × S | 2 | 13.81 | **<0.001** | 105.59 | **<0.001** | 77.28 | **<0.001** | 16.64 | **<0.001** |

The significant effects (*P* < 0.05) are shown in bold.

**Table S4 Results of linear mixed-effects model showing the effects of habitats (*n* = 3), species (*n* = 2), and their interactions on the uptakes of NO_3_^-^, NH_4_^+^ and total dissolved inorganic nitrogen (DIN) existing in soil, and the ratio of NO_3_^-^ to NH_4_^+^ aborbed by the plant in each quadrat.**

| Effect | DF | Uptake of nitrogen per quadrat (mg m^-2^) | | | | | | Uptake ratio of NO_3_^-^ to NH_4_^+^ | |
| --- | --- | --- | --- | --- | --- | --- | --- | --- | --- |
|  |  | NO_3_^-^ | | NH_4_^+^ | | DIN | |  |  |
|  |  | *F* | *P* | *F* | *P* | *F* | *P* | *F* | *P* |
| Habitats (H) | 2 | 10.61 | **<0.001** | 64.76 | **<0.001** | 20.68 | **<0.001** | 167.21 | **<0.001** |
| Species (S) | 1 | 308.15 | **<0.001** | 444.12 | **<0.001** | 514.05 | **<0.001** | 60.93 | **<0.001** |
| H × S | 2 | 34.72 | **<0.001** | 78.12 | **<0.001** | 70.46 | **<0.001** | 35.21 | **<0.001** |

The significant effects (*P* < 0.05) are shown in bold.

**Table S5 Results of linear mixed-effects model showing the effects of habitats (*n* = 3), species (*n* = 2), and their interactions on the uptake rates of NO_3_^-^, NH_4_^+^ and total dissolved inorganic nitrogen (DIN) existing in soil, and the uptake rate ratio of NO_3_^-^ to NH_4_^+^.**

| Effect | DF | Uptake rate of nitrogen (mg N kg^-1^ soil dw) | | | | | | Uptake rate ratio of NO_3_^-^ to NH_4_^+^ | |
| --- | --- | --- | --- | --- | --- | --- | --- | --- | --- |
|  |  | NO_3_^-^ | | NH_4_^+^ | | DIN | |  |  |
|  |  | *F* | *P* | *F* | *P* | *F* | *P* | *F* | *P* |
| Habitats (H) | 2 | 16.59 | **<0.001** | 70.72 | **<0.001** | 21.83 | **<0.001** | 166.24 | **<0.001** |
| Species (S) | 1 | 23.38 | **<0.001** | 96.90 | **<0.001** | 94.51 | **<0.001** | 61.25 | **<0.001** |
| H × S | 2 | 24.42 | **<0.001** | 64.86 | **<0.001** | 55.58 | **<0.001** | 34.73 | **<0.001** |

The significant effects (*P* < 0.05) are shown in bold.

**Table S6 Results of linear mixed-effects model showing the effects of habitats (*n* = 3), species (*n* = 2), and their interactions on the proportional contributions of NO_3_^-^ and NH_4_^+^ to plant N.**

|  |  | *f*_NO3_^-^ | |  | *f*_NH4_^+^ | |
| --- | --- | --- | --- | --- | --- | --- |
|  |  | *F* | *P* |  | *F* | *P* |
| Habitats (H) | 2 | 168.44 | **<0.001** |  | 119.99 | **<0.001** |
| Species (S) | 1 | 153.71 | **<0.001** |  | 11.82 | **<0.01** |
| H × S | 2 | 57.47 | **<0.001** |  | 15.46 | **<0.001** |

The significant effects (*P* < 0.05) are shown in bold.

**Table S7 Results of linear mixed-effects model showing the effects of habitats (*n* = 3), species (*n* = 2), and their interactions on N form preferences (*β*).**

|  |  | *β*_NO3_^-^ | |  | *β*_NH4_^+^ | |
| --- | --- | --- | --- | --- | --- | --- |
|  |  | *F* | *P* |  | *F* | *P* |
| Habitats (H) | 2 | 19.44 | **<0.001** |  | 19.44 | **<0.001** |
| Species (S) | 1 | 31.07 | **<0.001** |  | 31.07 | **<0.001** |
| H × S | 2 | 9.36 | **<0.001** |  | 9.36 | **<0.001** |

The significant effects (*P* < 0.05) are shown in bold.

**Table S8 Results of linear mixed-effects model showing the effects of habitats (*n* = 3), species (*n* = 2), and their interactions on the percentage similarities between plant uptake pattern of different N forms and their availability pattern in rhizosphere soil.**

| Effect | DF | *F* | *P* |
| --- | --- | --- | --- |
| Habitats (H) | 2 | 6.10 | **<0.01** |
| Species (S) | 1 | 18.81 | **<0.001** |
| H × S | 2 | 0.23 | 0.798 |

The significant effects (*P* < 0.05) are shown in bold.

**1.2 Supplementary Figures**

**Supplementary Figure 1.** Schematic sketch showing the method to label the plant with ^15^N using soil solution samplers

**Supplementary Figure 2.** Uptake rates of NO_3_^-^ (A), NH_4_^+^ (B) and total dissolved inorganic nitrogen (C) existing in soil, and the rate ratio of NO_3_^-^ to NH_4_^+^ (D) absorbed by *Solidago canadensis* (closed bars) and *Artemisia lavanduiaefolia* (open bars) in different habitats. NN, nitrate nitrogen; AN, ammonium nitrogen; DIN, dissolved inorganic nitrogen. Mean ± SE (*n* = 12). Different upper- and lowercase letters indicate significant differences among habitats for *S. canadensis* and *A. lavanduiaefolia*, respectively (*P* < 0.05; one-way ANOVA); * indicates significant differences between the two species in the same habitat (*P* < 0.05; independent sample *t*-test).

**Supplementary Figure 3.** Relationships between root to shoot ratios versus total biomass for *Solidago canadensis* (A) and *Artemisia lavanduiaefolia* (B).

**Supplementary Figure 4.** Relationships between the contents of total dissolved inorganic nitrogen, NO_3_^-^ and NH_4_^+^ versus root to shoot ratios for *Solidago canadensis* (A-C) and *Artemisia lavanduiaefolia* (D-F).
